# Study on the causes of growth differences in three conifers after the rainy season in the Xiong’an New Area

**DOI:** 10.3389/fpls.2023.1176142

**Published:** 2023-07-04

**Authors:** Xin Ran, Shenqi Qiao, Yu Zhang, Xiaokuan Gao, Yuewei Du, Bingxiang Liu, Changming Ma, Hongxiang Mu

**Affiliations:** ^1^ Department of Forest Cultivation, College of Forestry, Hebei Agricultural University, Baoding, China; ^2^ College of Life Science, Hengshui University, Hengshui, Hebei, China; ^3^ Hebei Urban Forest Health Technology Innovation Center, Baoding, China

**Keywords:** soil water content, conifers, hypoxia, root morphology, root respiration, root activity

## Abstract

**Background:**

The implementation of the Millennium Forestry Plan was accompanied by growth discomfort exhibiting varying degrees of symptoms in some coniferous forests after the rainy season.

**Hypothesis:**

High soil water content affects the underground root growth and distribution characteristics of conifers, and the above-ground parts show corresponding variability. To determine the factors contributing to the significant growth disparities among the three conifers in Xiong’an New Area after the rainy season, we conducted a study investigating the growth characteristics of conifers. This study involved analyzing the external morphology of the plants, assessing leaf pigment content, measuring the root morphological index and root vigor, as well as respiratory characteristics, to evaluate the growth attributes of their root systems in a high soil moisture environment.

**Methods:**

In the “Millennium Forest” area of Xiong’an New Area, we selected three coniferous trees, *Pinus tabuliformis*, *Pinus bungeana* and *Pinus armandii*, and set up three standard sample plots for each conifer. The conifers were classified into 3 levels according to their growth performance (vigorous or suppressed), leaf condition (color change, wilting or not) and relevant grading criteria.

**Results:**

(1) The growth of the three conifers displayed discernible differences in external morphology. Moreover, a decrease in growth condition corresponded to a reduction in crown size, ground diameter, diameter at breast height, leaf length, and new growths. (2) The root biomass, length, surface area, and root volume of conifers growing N class were significantly reduced than those of L class conifers. Conifers with a higher proportion of root systems in the 40-60 cm soil layer experienced more severe stress. (3) The significant decline in root respiration and vigor among all three conifer growth classes (M and N) suggested that the root system was undergoing anoxic stress, particularly at a soil depth of 40-60 cm where root respiration and vigor were notably reduced. (4) The persistent anoxic stress created by long-term exposure to high soil moisture content primarily impacted *P. armandii* to a greater extent than *P. tabuliformis* and *P. bungeana*. Additionally, the transporting and absorbing root ratios varied among conifers with differing growth conditions. The long-term high moisture environment also caused partial death of absorbing roots, which played a key role in the observed differences in growth. (5) As the soil depth increases, the soil water content increases accordingly. Plants with more root distribution in the deeper soil layers grow worse than those distributed in the top soil layers. Soil water content is related to aeration, root distribution, growth and growth of above-ground parts. The variability of root distribution and growth led to the differentiation of the growth of the above-ground part of the plant in terms of external morphology, which inhibited the overall plant growth. The results of the study provide a theoretical basis for the cultivation and management of three conifers in high soil moisture environments.

## 1 Introduction

Water is essential for the growth of plants. However, if water content exceeds a certain limit, it may impede the absorption of oxygen by plant roots and negatively impact their normal growth and development, as well as their ability to absorb nutrients (Mu et al., 2022). Excessive water in soil can result in secondary stress, which can cause significant harm to plants. When there is a lack of oxygen in soil, it can adversely affect the absorption and supply of oxygen to roots, leading to anaerobic respiration and an accumulation of harmful substances like acetaldehyde and ethanol (Pai-Hsiang and Chian-Ho, 1996; Gao et al., 2022). The root system is not only vital for the growth of plants but also for their overall development. The development and extension of plant roots in soil are influenced by several factors, including water, fertilizer, air, heat, and others. For the root system to grow and develop properly, it requires the coordinated action of several factors. The distribution of plants and crop yield is significantly impacted by high soil water content, which has become a critical limiting factor due to global warming, extreme precipitation, rising groundwater levels, and unreasonable irrigation measures, among other factors. Given that the root system is in direct contact with the soil, it is particularly susceptible to environmental stress factors related to soil moisture content. Plants experience stress first and foremost in their root system, which then transfers to the shoot for exchange of materials and nutrients. The growth and nutrient levels of plants are directly influenced by the status of their root system. Therefore, studying the root system of plants in adverse environments is critical to understanding how they respond to stress and identifying ways to mitigate the detrimental effects.

The Xiong’an New Area is located in the Baiyangdian basin, with water resources totaling approximately 173 million m^3^, of which the proportion of underground water resources is more than 90% (Liu, 2019; Xu et al., 2019). It is a water-rich area with a high groundwater table, which leads to high soil water content in the rainy season. The goal of the “Millennium Forest” project in the Xiong’an New Area is to establish a natural forest that incorporates both landscape and recreational elements to achieve an overarching visual effect of year-round greenery, with blossoms throughout three seasons. To enhance the aesthetic quality and overall value of the forest, the percentage of evergreen trees in the area will be limited to a range of 20% to 30%. The primary evergreen trees planted in the area include *Pinus tabuliformis*, *Pinus bungeana*, *Pinus armandii*, *Platycladus orientalis*, and *Sabina chinensis*. *Pinus armandii*, belongs to the Cembra Spach sect of the genus Pinus and is characterized by its rapid growth, remarkable ability to adapt to changing environments, and ecological functions related to water and soil conservation. As such, it has become a critical species in areas that receive little rainfall, particularly in the drier regions of the western area (He et al., 2020; Yao et al., 2021). The unique *Pinus bungeana* is the only three-needled pine tree found in East Asia, characterized by a remarkable ability to tolerate dry conditions, thrive under direct sunlight, and grow amidst nutrient-poor soils and cold climates. Further, it has been observed that *Pinus bungeana* grows exceptionally well in relatively cool zones with deep, fertile, and calcium-rich soils (Guo et al., 2021; Duo and Gao, 2022). *Pinus tabuliformis* is a heliophilic species, preferring a cold, drought-prone, and infertile soil. It is suitable for deep, loose, and acidic soil that is well-drained and moist. It has developed roots and strong soil and water conservation ability, so it is one of the best pioneer tree species for mountain shelter forests and timber forests (Du et al., 2020; Wang, 2020).

With the implementation of the Millennium Forest Project, some coniferous forests have experienced different degrees of growth discomfort during the rainy season, which has had a great impact on the normal ecological function of the Millennium Forest. At present, there are few studies on coniferous forests in the Xiong’an New Area. *Pinus tabuliformis*, *Pinus bungeana*, and *Pinus armandii*, as the main conifers planted in the Xiong’an New Area, have shown an inadaptable growth phenomenon after the rainy season. We hypothesize that the higher soil moisture content in Xiong’an New Area affects the root growth and distribution of conifers, leading to differences in their above-ground growth. The aim of this study was to investigate the factors influencing the growth of conifers by assessing the external morphology, root growth status, root respiration, and overall vigor of three conifer species. The objective was to explore the reasons for growth variations in the different conifers within the Xiong’an New Area. The findings of this study provide a theoretical foundation for the selection of conifer species, the implementation of land preparation measures, and the application of mixed-species within the region. Additionally, this study offers a scientific basis for managing the “Millennium Forest” in an efficient and sustainable manner in the Xiong’an New Area.

## 2 Materials and methods

### 2.1 Experimental materials

The Located in the hinterland of Beijing, Tianjin, and Baoding, the Xiong’an New Area mostly covers the administrative areas of Xiong’an, Rongcheng, and Anxin counties in Hebei Province (including Baiyangdian Lake), with a planned area of approximately 1770 km^2^.The study area has a warm temperate monsoon continental climate with four distinct seasons. The annual average temperature here was 11.9 °C, the hottest monthly average temperature is 26.1 °C, the coolest monthly average temperature is -4.9 °C, the extreme maximum temperature reaches 40.9 °C, and the extreme minimum temperature is -21.5 °C. The annual sunshine is 2685 h; the annual average rainfall of 523 mm is mainly concentrated from June to September, accounting for 80%; the northerly wind has a maximum annual average wind speed of 2.1 m·s^-1^ throughout the year; and the frost-free period is 191 days. Low temperature, high temperature, strong wind, and rainy weather are the main limiting factors affecting the growth of tree species (Yi, 2021). The basic physical and chemical properties of soil in the study area are as follows: organic matter, 3.57 (g·kg^-1^); alkaline nitrogen, 125.18 (mg·kg^-1^); total nitrogen, 0.19 (g·kg^-1^); total phosphorus, 0.63 (g·kg^-1^); available phosphorus, 116.02 (mg·kg^-1^); pH 7.5 - 8.1.

### 2.2 Experimental design

In August 2022, three conifers (Pinus tabuliformis, Pinus bungeana, Pinus armandii) were selected from “Millennium Xiulin” aera (E116°2′19″, N38°59′40″) in the Xiong’an New Area. Then, we set three 20m×30m standard quadrats (9 quadrats in total). The plant growth of the three conifers was investigated. Some scholars took a 20% proportion of damaged leaves as the classification standard, which was then further divided into five levels (Ji et al., 2015). Other scholars took the proportion of dead leaves as the classification standard, and divided it into five levels: 0%, 0-20%, 20-50%, 50-70%, and > 70% (Meng et al., 2018). According to the growth performance (vigorous or inhibited growth), leaf status (color change, wilting or not), and grading standards of relevant researchers (Ji et al., 2015; Meng et al., 2018), a comprehensive evaluation was made by us. Plants were grouped into 3 levels following the severity of stress symptoms: L Level: vigorous growth; dark leaves; no discoloration and death. M Level: the growth was average; the leaf color was basically normal or light; slightly yellow; and the leaf mortality was less than 40%. N Level: growth is inhibited; leaves are wilted; curled or yellow; drooping; 40% ≤ leaf death. According to the stress levels (L, M, N), the 3 conifer types with different growth states were selected and numbered in 9 standard plots (each conifer type had 3 growth levels, 3 trees were selected for each level for a total of 27 trees) and we repeated the experiment 3 times. As can be seen from **Table 1**, the initial tree heights of *P. armandii*, *P. bungeana* and *P. tabuliformis* were 3.45m, 1.83m, and 1.84m respectively. Their initial crown widths were 1.71m, 1.18m, and 1.35m, respectively. Their initial ground diameters were 6.77cm, 3.73cm, and 7.23cm, respectively.

**Table 1:** Basic information about the three selected conifers.

Tree species	Place of origin	Time of planting	Initial tree height (m)	Initial crown width (m)	Initial ground diameter (cm)	Growth level	The proportion(%)	Abbreviation
*Pinus armandii*	Anguo City Tianhe seedling farm	December 2017	3.45 ± 0.11	1.71 ± 0.09	6.77 ± 0.31	L	12.29 ± 1.5	Pa-L
						M	49.26 ± 2.33	Pa-M
						N	38.44 ± 3.83	Pa-N
*Pinus bungeana*	Yi County South Liuquan Green Garden nursery	March 2018	1.83 ± 0.02	1.18 ± 0.08	3.73 ± 0.42	L	40.06 ± 1.53	Pb-L
						M	36.03 ± 2.22	Pb-M
						N	23.91 ± 2.58	Pb-N
*Pinus tabuliformis*	Manchu Mongolian autonomous county paddock base	March 2018	1.84 ± 0.05	1.35 ± 0.05	7.23 ± 1.23	L	68.87 ± 1.00	Pt-L
						M	20.6 ± 1.32	Pt-M
						N	10.53 ± 1.02	Pt-N

### 2.3 Materials and methods

#### 2.3.1 Determination of tree growth indicators

The growth state of the sample tree was photographed. The leaf morphology was scanned by Win-RHIZO software. The new shoot and leaf length were measured with a straightedge. The tree height was measured with an SRC-1-30 quasi-continuous variable range altimeter (Harbin Optical Instrument Factory LTD, China). The diameter at breast height and the ground diameter were measured with a tape measure.

#### 2.3.2 Determination of soil indexes

During August 2022, three sampling points were randomly chosen from the three standard coniferous sample plots. The soil layers from 0-20 cm, 20-40 cm, and 40-60 cm were collected by tapping the ring knife using a rubber hammer. The soil was excavated and evenly leveled to ensure that the soil volume matched the ring knife’s volume precisely. The ring knife soil samples were brought back to the laboratory with damping measures. Half soil samples were measured by drying method (Guo et al., 2022). The particle size was measured by a Bettersize 2000 laser particle size (Dandong Baxter Instrument Company), and the soil texture was analyzed by international texture classification. The maximum field moisture capacity and soil bulk density and porosity were measured by the ring knife method.

The rest of the soil samples were used to measure soil permeability. We removed the top cover of the ring knife with undisturbed soil samples, put it in a flat-bottom basin paved with quartz sand, and slowly injected clean water into the basin along the sidewall of the flat bottom basin. When the water surface falls below 2 mm of the upper edge of the ring knife, the injection of water was stopped. When a layer of water film appeared on the soil surface, it indicated that the soil was saturated. We covered the top cover of the ring knife that was soaked in water, removed the bottom cover, and wiped off the water on the sidewall of the ring knife with a dry towel. After 6 h of placement, the pressure gauge method was used to repeatedly measure the soil aeration inside the ring cutter 3 times, and then, the ring cutter was placed again at 40°C for 4 h (Yi, 2009). Following that, the ring knife was sealed at the top and bottom using a sealing film, and left for 6 hours before being removed for weighing to measure the aeration (Cheng et al., 2018). The above operations were repeated in turn until the ring cutter’s weight difference was within 0.1 g, and the experimental data were recorded. The soil aeration was calculated according to a formula. The correlation equation was established by taking the soil water content at different depths as the independent variable and the soil aeration coefficient as the dependent variable.

#### 2.3.3 Determination of root index

Sampling was carried out around the trunk of the sample tree, with a radius of 20cm (**Figure S1**). The southeast side of the tree was selected and samples were taken from inside out, starting from A (20-40cm), B (40-60cm), C (60-80cm), D (80-100cm), E (100-120cm), F (120-140cm), G (140-160cm), H (160-180cm), and I (180-200cm), till the roots were no longer visible. The vertical depth was categorized into three layers: 0-20cm, 20-40cm, and 40-60cm. The root system was carefully extracted from the soil samples using a 0.15mm mesh and placed into self-sealing bags, which were labeled and kept refrigerated at 4°C until further analysis.

In the laboratory, the roots were washed several times with clean water, followed by the removal of all non-sample tree roots (including other plant roots). After selecting the roots, we placed them into labeled envelopes for further analysis. The Win-RHIZO software was used to measure the characteristic parameters of the fresh fine roots, including root length (Root Length, cm), root surface area (Surface Area, cm^2^), and root volume (Root Volume, cm^3^).

Various classifications of root diameters have been documented in the literature for different plant species (Wang et al., 2005; Guo et al., 2008). Gu et al. (2017) and Xie et al. (2014) defined root systems with diameters ≤ 0.5 mm and 0.5-2.0 mm as absorbing roots and transporting roots respectively, which are collectively called fine roots. Other authors (Hendrick and Pregitzer, 1993; Fogel, 1983; Fujita et al., 2021) have also reported that roots with diameters< 2 mm or< 5 mm are defined as fine roots, and some works have even reported that roots with smaller diameters, such as 0.5 mm or smaller, should be defined as fine roots (Pregitzer et al., 2002). Liu (2007) divided the roots of *Pinus tabuliformis* and *Pinus bungeana* into four levels according to their diameters (<1 mm, 1-2 mm, 2-5 mm and >5 mm). Based on the actual situation and the literatures, the diameter of root system was divided into I(<2mm), II(2-4 mm), III(4-6 mm), and IV(> 6 mm), in which ≤ 6 mm was an absorbing root and > 6mm was a transporting root. After scanning, the roots were dried at 80 °C to constant weight, and the biomass was measured.

Root respiration of fresh roots was measured by the alkali absorption method (Song et al., 2020) (**Figure S2**). A centrifuge tube was put into a large round bottle, and 5mL 0.4 mol·L^-1^ NaOH standard solution was added to a large round bottle. In total, 0.5 g of the root system was put into the large-cover centrifugal tube and covered it well. CO_2_ was released by root respiration sinks naturally and was absorbed by lye for 2 h at a certain temperature. The centrifuge tube was then removed. The 5 mL of saturated barium chloride solution and 2 drops of phenolphthalein (0.1ml in total) were added to a large, round bottle. Then, 0.1 mol·L^-1^ oxalic acid standard solution titrated the solution in the round bottle, and the amount of oxalic acid standard solution was recorded. The same method was used to perform blank titration. Root respiration was calculated as follows:


$$\text{Root respiration }({\text{CO}}_{2}\text{mg}/\left(\text{kg}\cdot \text{h}\right)=\left(\text{V}1-\text{V}2\right)\cdot 44\text{C}/\left(\text{m}\cdot \text{t}\right)$$

V1= Oxalic acid dosage for blank titration, mL; V2 = Oxalic acid dosage for sample titration, mL; C = Molar concentration of H_2_C_2_O_4_, mol·L-1; M = Quality of sample, Kg; t = Measured time, h.

The TTC method was used to measure root activity, with specific reference to plant physiology experimental guidance (Li and Zhang, 2016).

#### 2.3.4 Determination of photosynthetic pigments

The contents of chlorophyll a, chlorophyll b, total chlorophyll and carotenoids of three coniferous trees with different grading standards were determined with an acetone, ethanol, and water mixture (Hao, 2015). Three leaves (upper, middle and lower) from three parts of each tree were randomly collected. Then, the leaf samples were put into a refrigerator at 4°C and taken back to the laboratory for immediate determination. 0.1g of leaves were finely sliced and placed into the test tube. Then, 10 mL of the mixture (acetone: ethanol: water =4.5:4.5:1) was added to the test tube. The tube seal was placed in the dark overnight until the leaf filaments changed from green to white. Acetone-ethanol-water mixture (acetone: ethanol: water =4.5: 4.5: 1) was used as the control solution. The optical density of the extract at 663 nm, 645 nm, and 440 nm was determined by spectrophotometer. The contents of chlorophyll a, chlorophyll b, total chlorophyll, and carotenoid were calculated by the following formula:


$$\text{The content of chlorophyll a}\left(\text{mg}/\text{g}\right)=12.71\cdot {\text{OD}}_{663}-2.59{\text{OD}}_{645}$$

$$\text{The content of chlorophyll b}\left(\text{mg}/\text{g}\right)=22.88{\text{OD}}_{645}-4.67{\text{OD}}_{663}$$

$$\text{The content of total chlorophyll}\left(\text{mg}/\text{g}\right)=20.29{\text{OD}}_{645}+8.04{\text{OD}}_{663}$$

$$\text{The content of Carotenoid}\left(\text{mg}/\text{g}\right)=0.1\cdot \left(5.695{\text{OD}}_{440}-0.268\cdot \text{the content of total chlorophyll}\right)$$

### 2.4 Data processing

Excel and Origin were used to fit the response curve data. SPSS 18.0 data processing software was used for statistical analysis, and the least significant difference (LSD) method was used to check the significance of the difference when the P value was less than 0.05.

## 3 Results

### 3.1 Changes in soil water content and aeration at different depths

**Figure 1:**
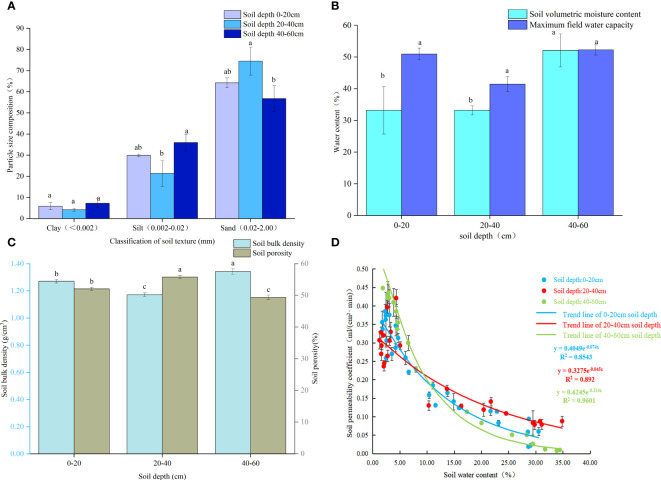
Soil propriities: **(A)** Soil composition; **(B)** Soil moisture content and field capacity; **(C)** Soil bulk density and porosity; **(D)** Relations among soil water content and permeability coefficient. Note: Different small letters in the same column meant significant difference at 0.05 level among soil depth.

The proportion of soil particle size varied with soil depth (**Figure 1A**). The greatest proportion of clay content was recorded at a depth of 40-60 cm with a value of 7.25%, followed by 5.89% at 0-20 cm depth and 4.15% at 20-40 cm depth. With increasing soil depth, the proportion of silt was 29.88%, 21.38%, and 35.97%. The highest proportion of sand in the 20-40 cm soil was 74.47%, the lowest in the 40-60 cm soil was 56.78%, and the middle in the 0-20 cm soil was 64.23%. According to the soil texture, the soil in the sample plots was sandy loam. The soil water content increased with increasing soil depth(**Figure 1B**). At soil depths of 0-20 cm, 20-40 cm, and 40-60 cm respectively, the soil water content was measured to be 32.20%, 33.21%, and 52.09%. With increasing soil depth, the maximum field capacities of the soil were 50.94%, 41.44%, and 52.25%, respectively. The soil water content in the vertical direction accounted for 63.21%, 80.13%, and 99.69% of the field water capacity, respectively. The soil porosity was 20-40 cm > 0-20 cm > 40-60 cm at different soil depths, respectively (**Figure 1C**). The bulk density of 40-60 cm soil was 1.34 g·cm^-3^ higher than the 1.27 g·cm^-3^ and 1.17 g·cm^-3^ of the 0-20 cm and 20-40 cm soils, respectively. With increasing soil water content, soil aeration decreased (**Figure 1D**). At 0-20 cm, the correlation between the water content and air permeability was the lowest (R^2 = ^0.8543). At 20-40 cm, the correlation between water content and air permeability was R^2 = ^0.892. The highest correlation between soil water content and the soil permeability coefficient was found at the 40-60 cm soil depth (R^2 = ^0.9601).

### 3.2 Changes in phenotypic growth of the three conifers

**Figure 2:**
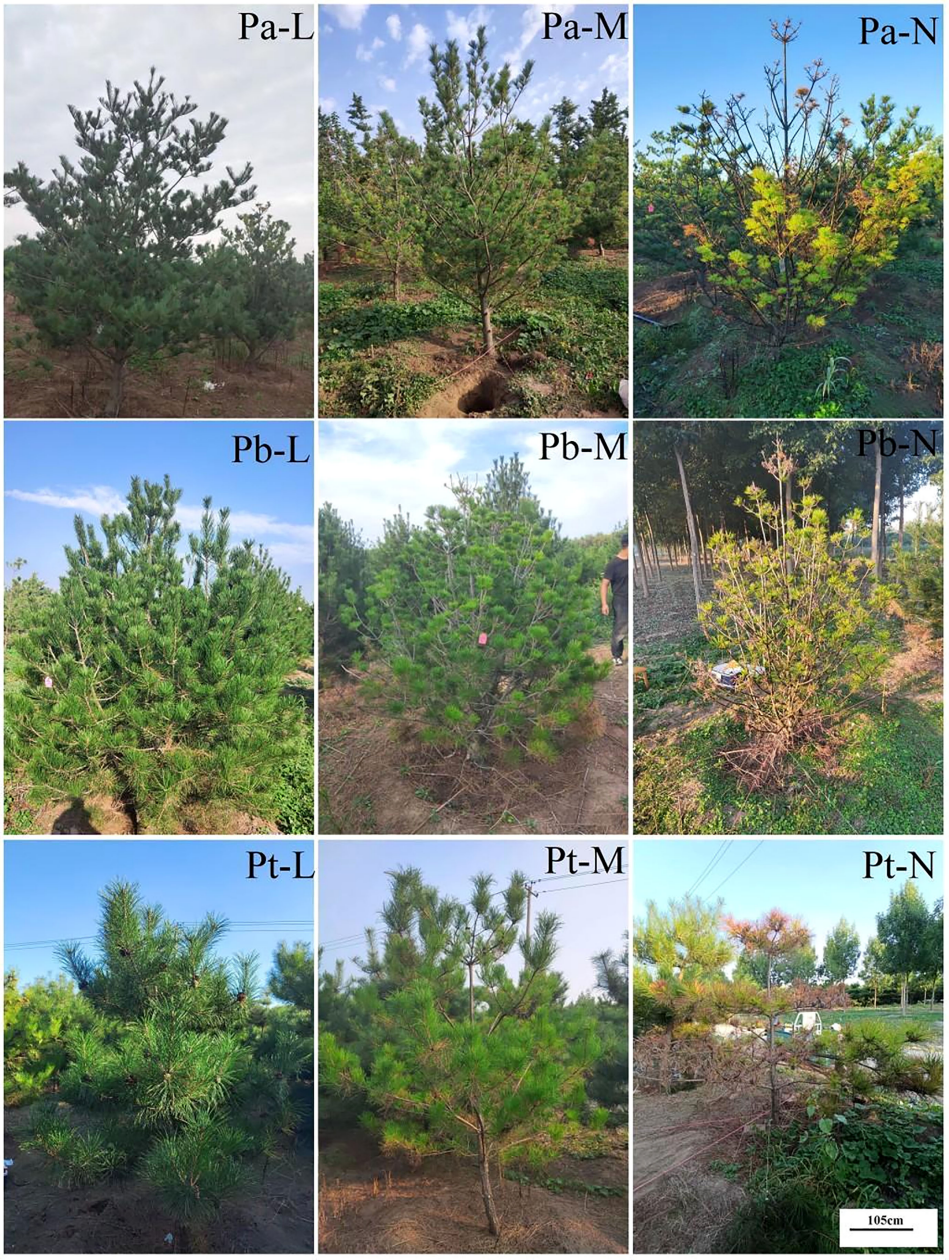
Phenotype of three conifers.

**Figure 3:**
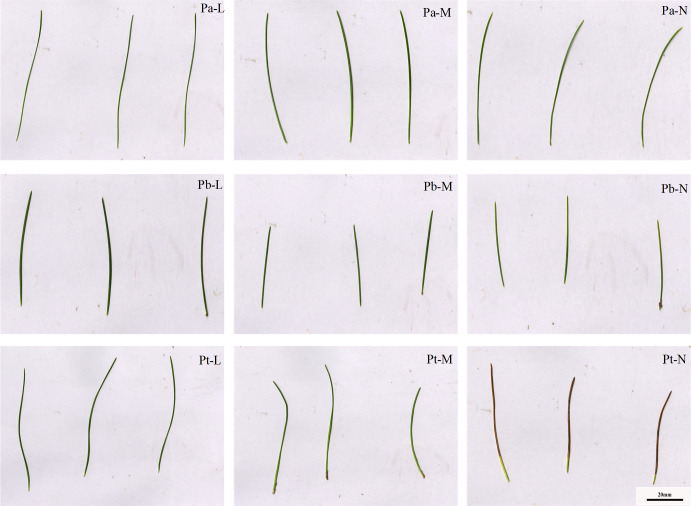
Leaf phenotypes of three conifers.

The L-level conifers were dark green; the leaves of the M-level conifers were light green; and the leaves of the N-level conifers showed senescence, yellowing, and even wilting at the top of the leaves (**Figures 2**, **3**). In addition, and the length of the conifers became shorter with the deterioration of tree species growth (**Table 2**). The leaf length of *P. tabuliformis* was longer than that of *P. bungeana*; the shortest leaf length of *P. armandii* needles were, in order, Pa-N< Pa-M< Pa-L, and the difference was significant (P< 0.05). The leaf lengths of N-level and L-level needles decreased by 34.68%, 30.32%, and 32.57% respectively, for *P. armandii*, *P. tabulaeformis* and *P. bungeana*. There was a gradual reduction in the length of shoots with the increasing severity of plant damage. Compared with Pa-N, the shoot growth of Pa-L, and Pa-M increased by 219.32% and 59.45%, respectively. Compared with Pb-N, Pb-L and Pb-M increased by 338.62% and 136.32%, respectively.

**Table 2:** Growth phenotype of three conifers under different growth conditions.

Abbreviation	Ground diameter (cm)	Breast height diameter (cm)	Tree height (cm)	Crown diameter (cm)	Leaf length (cm)	Shoots growth (cm)
Pa-L	10.23 ± 0.12a	6.60 ± 0.32a	3.76 ± 0.05a	3.15 ± 0.03a	8.76 ± 0.24a	15.04 ± 1.37a
Pa-M	9.40 ± 0.10b	4.97 ± 0.34b	3.23 ± 0.02b	2.82 ± 0.03b	7.48 ± 0.18b	7.51 ± 0.38b
Pa-N	9.20 ± 0.12b	4.27 ± 0.03c	3.07 ± 0.04c	2.41 ± 0.03c	5.86 ± 0.10c	4.71 ± 0.80c
Pb-L	10.30 ± 0.30a	2.73 ± 0.20a	2.81 ± 0.03a	2.16 ± 0.02a	6.63 ± 0.18a	19.08 ± 2.17a
Pb-M	8.40 ± 0.57b	2.00 ± 0.21b	2.18 ± 0.01b	1.81 ± 0.02b	5.00 ± 0.05b	10.28 ± 0.86b
Pb-N	7.57 ± 0.23c	1.97 ± 0.09c	1.94 ± 0.06c	1.66 ± 0.04c	4.47 ± 0.10c	4.35 ± 0.32c
Pt-L	9.83 ± 0.22a	3.87 ± 0.39a	2.68 ± 0.03a	2.52 ± 0.02a	15.17 ± 0.22a	25.25 ± 1.50a
Pt-M	7.40 ± 0.46b	3.07 ± 0.26b	2.48 ± 0.04b	2.36 ± 0.03b	8.56 ± 0.49b	16.42 ± 2.16b
Pt-N	7.07 ± 0.29c	2.63 ± 0.39c	2.14 ± 0.02c	2.12 ± 0.07c	10.57 ± 0.41c	9.80 ± 1.03c

Some conifers showed symptoms of injury (**Table 2**). The tree height of *P. armandii* and *P. bungeana* increased with the aggravation of damage in the M- and N- levels, while the index of crown width decreased with the aggravation of damage. The tree height and crown width of *P. tabulaeformis* showed that Pt-L > Pt-M > Pt-N. The diameter at breast height and the ground diameter of the three conifers exhibited a decline with the worsening of damage severity.

Different small letters in the same column meant significant difference at 0.05 level among the same tree species. The same below.

### 3.3 Changes in chlorophyll a, b and carotene contents of three conifers

There was a gradual decrease in the photosynthetic pigment content in the three coniferous trees with increasing stress levels. The order of chlorophyll a content from high to low was L>M>N (**Table 3**). Compared with the growth N-level growth, the chlorophyll a content in L-level *P. armandii*, *P. bungeana* and *P. tabulaeformis* increased significantly by 1.32, 0.97 and 2.34 times, respectively. Compared with the growth N level growth, the chlorophyll b content in Pa-M, Pb-M, and Pt-M increased by 5.83%, 4.64% and 2.08% respectively. The carotenoid content of the three coniferous trees under different growth conditions was significantly different. Compared with growth M-level growth, the carotenoid content of the growth N-level (Pb-N, Pa-N, Pt-N) of the three coniferous trees decreased by 23.57%, 31.05%, and 27.56%, respectively.

**Table 3:** Photosynthetic pigment concentrations of three conifers.

Abbreviation	Chlorophyll a (mg·g^-1^)	Chlorophyll b (mg·g^-1^)	Carotenoid (mg·g^-1^)
Pa-L	0.77 ± 0.02a	0.45 ± 0.03a	0.22 ± 0.00a
Pa-M	0.54 ± 0.00b	0.35 ± 0.00b	0.14 ± 0.00b
Pa-N	0.33 ± 0.03c	0.34 ± 0.05b	0.10 ± 0.01c
Pb-L	0.60 ± 0.00a	0.43 ± 0.01a	0.13 ± 0.00a
Pb-M	0.49 ± 0.00b	0.42 ± 0.00b	0.12 ± 0.00b
Pb-N	0.31 ± 0.00c	0.40 ± 0.00c	0.08 ± 0.00c
Pt-L	0.83 ± 0.06a	0.56 ± 0.02a	0.20 ± 0.00a
Pt-M	0.39 ± 0.04b	0.42 ± 0.03b	0.09 ± 0.01b
Pt-N	0.25 ± 0.00c	0.43 ± 0.00b	0.07 ± 0.00c

### 3.4 Changes in root morphology of three conifers

#### 3.4.1 Root biomass

**Figure 4:**
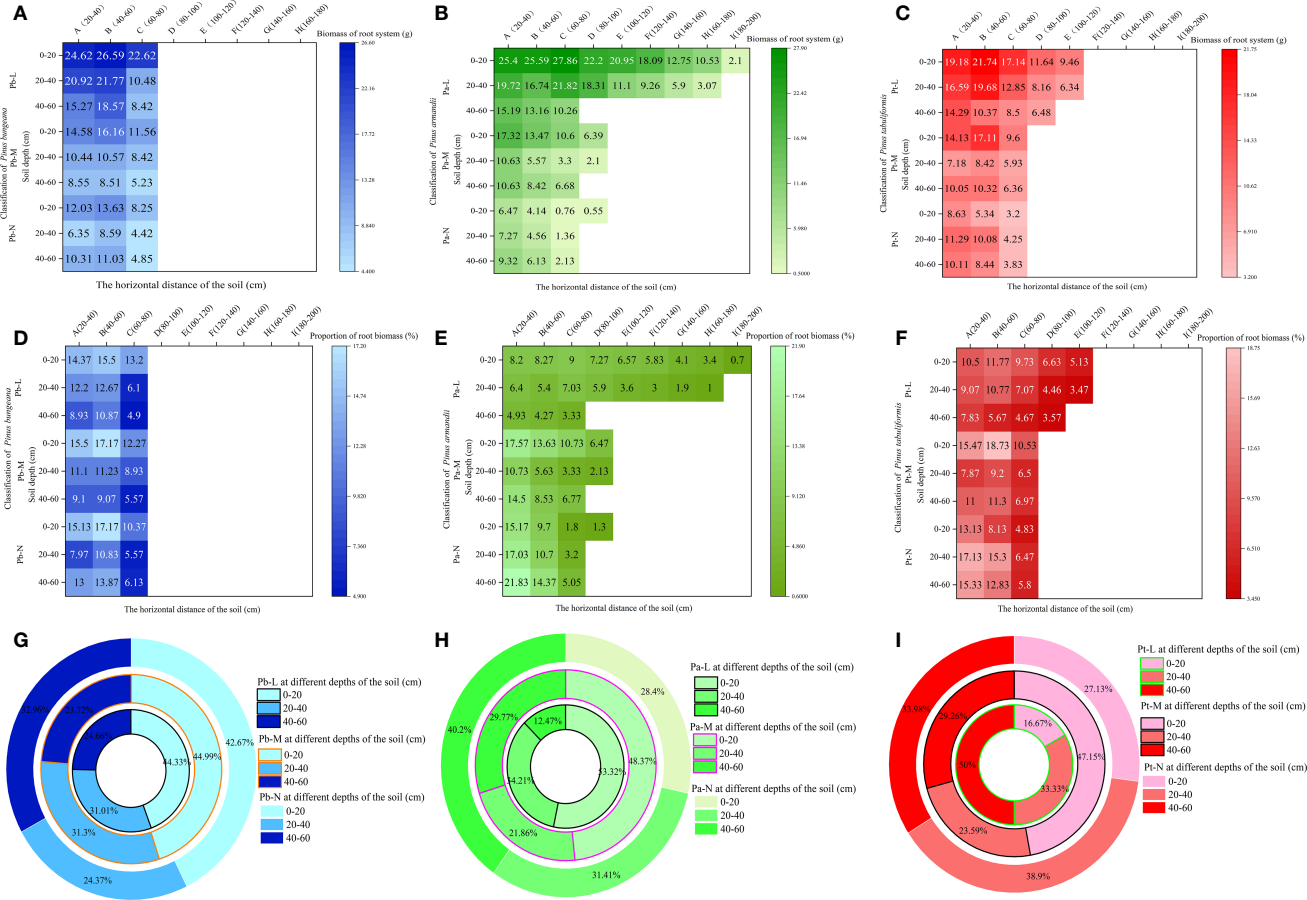
Effect of high soil water content on root biomass of three conifers. Horizontal and vertical distribution of root biomass of three conifers: **(A)** *P. bungeana*; **(B)** *P. armandii*; **(C)** P. tabulaeformis; The proportion of biomass distribution of three conifers: **(D)** *P. bungeana*; **(E)** *P. armandii*; **(F)** *P. tabulaeformis*. Vertical distribution of root biomass of three conifers: **(G)** *P. bungeana*; **(H)** *P. armandii*; **(I)** *P. tabulaeformis*.

Root biomass is an indicator reflecting the growth ability of the root system. A higher biomass indicates stronger nutrient and water absorption capacity of the roots. Root biomass can adapt to environmental changes, and the horizontal and vertical distribution of biomass directly reflect the distribution characteristics of the root system (Xiao et al., 2018). The horizontal range of the root distribution of *P. bungeana* was 20-80 cm (**Figure 4A**). The proportion of root biomass in Pb-L and Pb-M increased initially with the expansion of the horizontal range, but later exhibited a decline. Moreover, the root biomass of these trees significantly decreased with an increase in soil depth (**Figure 4D**). There was a significant upward trend in the proportion of Pb-N root biomass as the soil depth increased. However, beyond a certain soil depth, the proportion decreased notably. The proportion of root biomass in horizontal layer A of Pb-L at 20-40 cm and 40-60 cm was 15.1% and 37.85% lower than that at 0-20 cm, respectively. The proportion of root biomass in horizontal layer A of Pb-M at 20-40 cm and 40-60 cm was 28.38% and 41.29% lower than that at 0-20 cm, respectively. The proportion of root biomass in horizontal layer A of Pb-N at 20-40 cm and 40-60 cm was 47.32% and 14.07% lower than that at 0-20 cm, respectively. At the depth of 20-40 cm, the proportion of root biomass of Pb-N and Pb-L was significantly lower than that of Pb-M; at 40-60 cm, it showed a significant increasing trend (**Figure 4G**).

The root system of Pa-L demonstrated the most extensive distribution in the topmost soil layer, 0-20 cm from the base of the tree. Notably, even at a distance of 180-200 cm from the tree base, there remained a notable distribution of root biomass, which measured 2.1g. At a depth of 20-40 cm, the root system was 160-180 cm away from the tree body. At a depth of 40-60 cm, it was only distributed 60-80 cm away from the tree body (**Figure 4B**). The proportion of the root biomass of Pa-L increased first and then decreased with the expansion of the horizontal range at a depth of 0-40 cm, and it significantly decreased with increasing soil depth (**Figure 4E**). The expanding horizontal range was associated with a decrease in the proportion of root biomass in both Pa-M and Pa-N. The proportion of the root biomass of *P. armandii* at 0-20 cm decreased significantly with the deterioration of growth: 53.32%, 48.37% and 28.4% respectively (**Figure 4H**). At 20-40 cm, the proportion of the root biomass of Pa-L, Pa-M and Pa-N was 34.21%, 21.86%, and 31.41%, respectively, showing a significant difference. There was a significant enhancement in the proportion of P. armandii root biomass observed at a depth of 40-60 cm, which was consistent with higher growth grading of the tree. The proportion of the root biomass of Pa-M at different soil depths was as follows: 0-20 cm>40-60 cm>20-40 cm. The proportion of Pa-N’s root biomass at different soil depths was as follows: 0-20 cm<20-40 cm<40-60 cm.

The Pt-L roots were still distributed 100-120 cm away from the tree body; the root distribution range of Pt-M and Pt-N was 60-80 cm at most (**Figure 4C**). With increasing soil depth, the proportion of the root biomass of Pt-L gradually decreased significantly (**Figure 4F**). The proportion of root biomass in Pt-L showed an initial rise followed by a decline as the horizontal distance from the tree’s base increased, at a depth between 0-40 cm. At a depth of 40-60 cm, it decreased with increasing horizontal distance. At different soil depths, the proportion of the root biomass of Pt-M first increased and then decreased with increasing horizontal distance. The proportion of root biomass of Pt-M at different soil depths was as follows: 0-20 cm>40-60 cm>20-40 cm. The proportion of Pt-N’s root biomass in different soil depths was as follows: 0-20 cm<40-60 cm<20-40 cm. With an increase in horizontal distance, there was a reduction in the proportion of root biomass observed in Pt-N. The root biomass of *P. tabulaeformis* at different soil depths and different growth states showed significant differences (**Figure 4I**): Pt-M>Pt-L>Pt-N at 0-20 cm, Pt-N>Pt-L>Pt-M at 20-40 cm, and Pt-L<Pt-M<Pt-N at 40-60 cm.

#### 3.4.2 Root length

**Figure 5:**
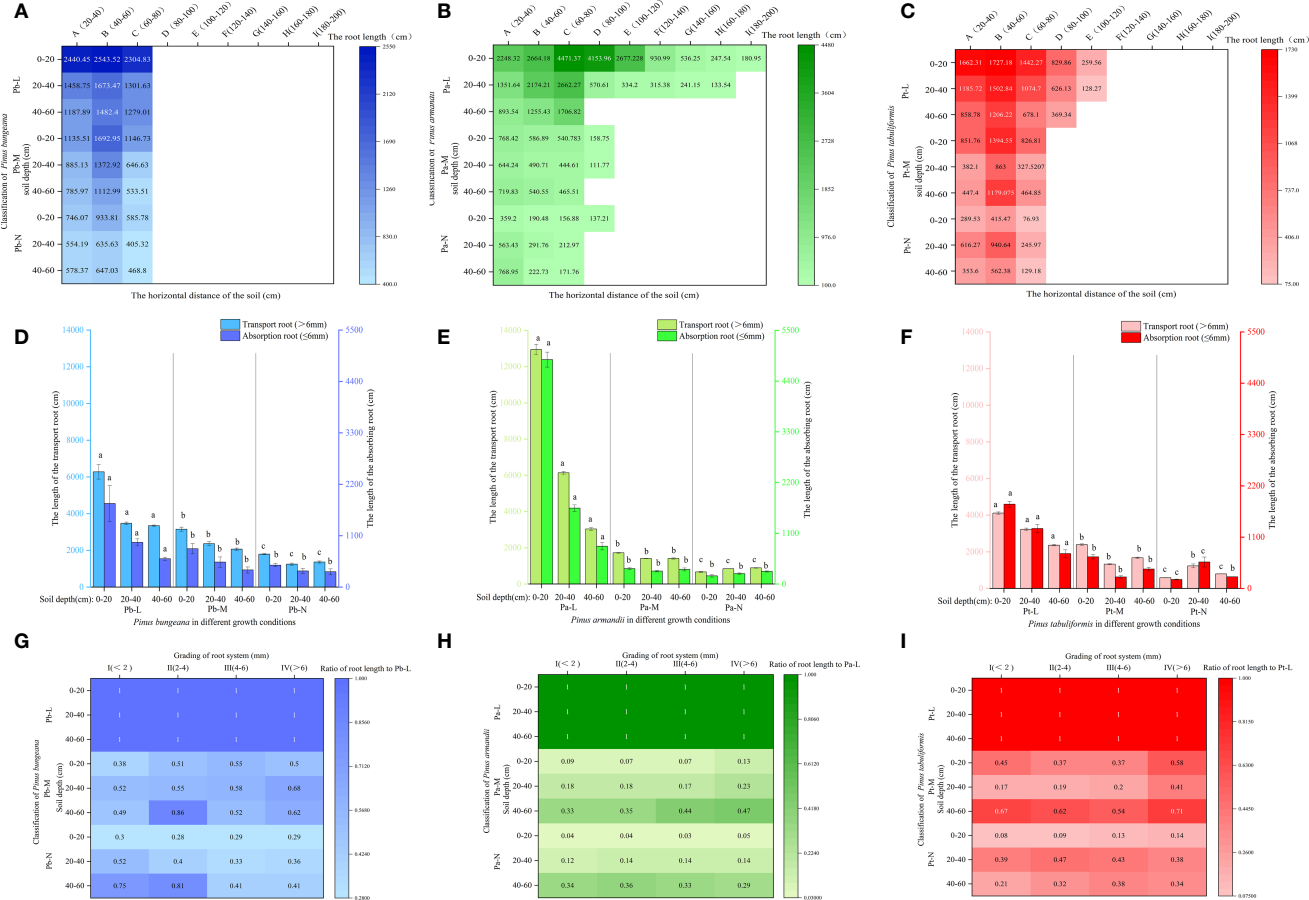
Effect of high soil water content on root length of three coniferous trees. Distribution of root length of three coniferous trees in the horizontal and vertical directions: **(A)** *P. bungeana*; **(B)** *P. armandii*; **(C)** P. tabulaeformis. The vertical distribution of the length of three coniferous trees grading roots: **(D)** *P. bungeana*; **(E)** *P. armandii*; **(F)** P. tabulaeformis. The ratio of grading root length of three coniferous trees to L-level in the vertical direction, respectively: **(G)** *P. bungeana*; **(H)** *P. armandii*; **(I)** *P. tabulaeformis*.

The length of a plant’s root system is an indicator of its developmental status, absorption capacity, and ability to withstand stress. When the soil depth was 0-20 cm, the root lengths of Pb-L, Pb-M, and Pb-N in horizontal range C were 9.3%, 32.26%, and 37.27% less than those in B, respectively (**Figure 5A**). The root length of Pa-N increased significantly with the increase of soil depth in the A-class range, but increased first and then decreased in the C-class range (**Figure 5B**). The root length of Pt-L decreased with increasing soil depth. Under different soil depths, the root length of Pt-M was different from that of Pt-L: 20-40 cm< 40-60 cm<0-20 cm. The Pt-N root length in different soil depths was as follows: 0-20 cm< 40-60 cm< 20-40 cm (**Figure 5C**). There was a gradual reduction in the lengths of both absorbing roots and transporting roots of the three conifers, in response to increasing soil depth. There was a decrease in the lengths of both absorbing roots and transporting roots of the three coniferous trees as growth levels increased at the same soil depth. Furthermore, we found that transporting roots were longer compared to the absorbing roots. (**Figures 5D–F**). Root surface area also showed the same trend as root volume. The ratio of root length to Pa-L of Pa-M and Pa-N increased with the increase of soil depth; The ratio of Pt-M root length to Pt-L decreased first and then increased with the increase of soil depth, while Pt-N increased first and then decreased (**Figures 5G–I**).

**Figure 6:**
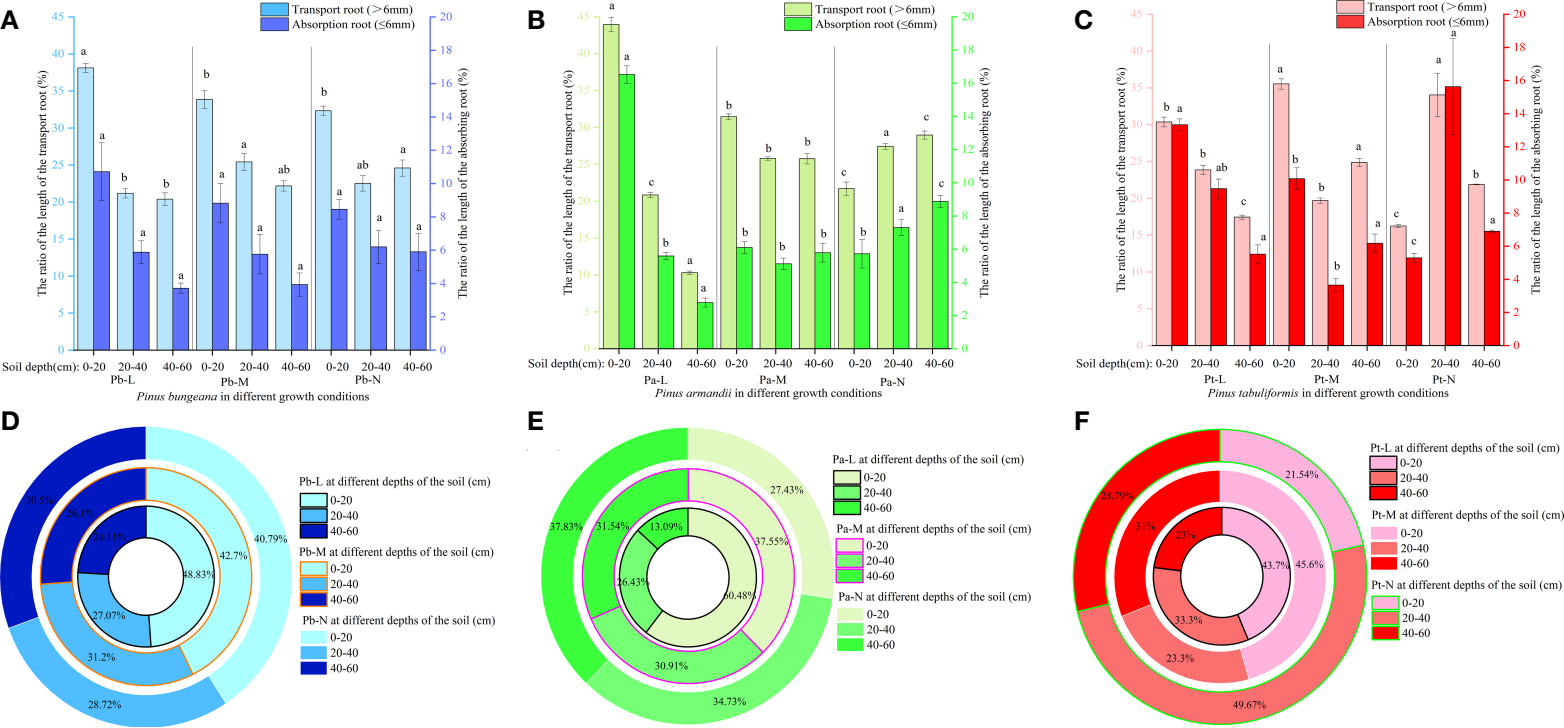
Effect of high soil water content on the proportion of root length of three conifers. The proportion distribution of root length of graded root: **(A)** *P. bungeana*; **(B)** *P. armandii*; **(C)** *P. tabulaeformis*. The proportion of root length in vertical direction: **(D)** *P. bungeana*; **(E)** *P. armandii*; **(F)** *P. tabulaeformis*.

With the increase in soil depth, the length proportion of the transport roots and absorption roots decreased gradually for Pb-L and Pb-M (**Figure 6A**). The distribution of Pb-N transport root length varied with soil depth, following the pattern of 0-20 cm > 40-60 cm > 20-40 cm. The proportion of transport root length decreased significantly with the deterioration of the growth of *P. armandii* at the 0-20 cm depths (**Figure 6B**). A contrasting trend was observed in the 20-40 cm and 40-60 cm soil layers, where there was a significant increase in the proportion of transport root length. At a depth of 0-20 cm, the root length proportion of transport and absorption root of Chinese pine in different growth states was significantly different, and Pt-N was the smallest (**Figure 6C**).

The distribution of root system length proportions differed amongst the three coniferous trees at varying soil depths. (**Figures 6D–F**). When the soil depth was 0-20 cm, the root length proportion of *P. bungeana*, *P. armandii*, and *P. tabulaeformis* from large to small, were Pb-L>Pb-M>Pb-N, Pa-L>Pa-M>Pa-N, and Pa-M>Pa-L>Pa-N. When the soil depth was 20-40 cm, the of root length proportions of *P. bungeana*, *P. armandii*, and *P. tabulaeformis*, from large to small,were Pa-M>Pa-N>Pa-L, Pa-N>Pa-M>Pa-L, and Pa-N>Pa-L>Pa-M. When the soil depth was 40-60 cm, the proportion of root length of *P. bungeana*, *P. armandii* and *P. tabulaeformis* from large to small was: Pa-N>Pa-M>Pa-L, Pa-N>Pa-M>Pa-L, Pa-M>Pa-N>Pa-L. At different levels of stratification, the root surface area of the three coniferous trees exhibited a similar trend to that of the root length.

#### 3.4.3 Root surface area

**Figure 7:**
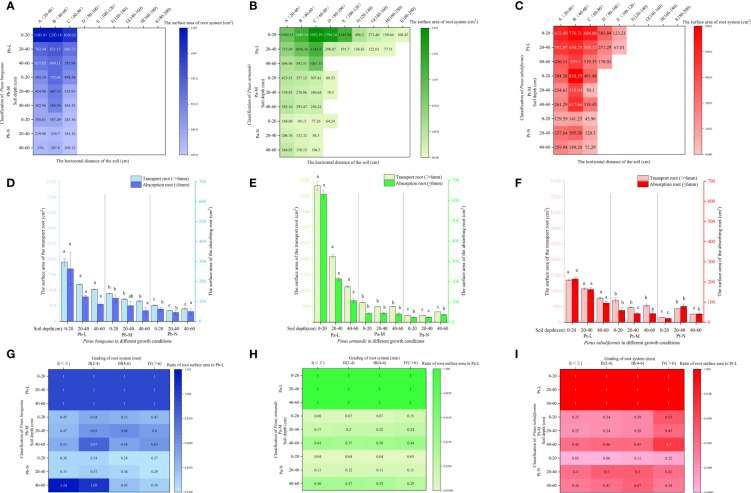
Effect of high soil water content on root surface area of three coniferous trees. Distribution of root surface area of three coniferous trees in the horizontal and vertical directions: **(A)** *P. bungeana*; **(B)** *P. armandii*; **(C)** *P. tabulaeformis*. The vertical distribution of the surface area of three coniferous trees grading roots: **(D)** *P. bungeana*; **(E)** *P. armandii*; **(F)** *P. tabulaeformis*. The ratio of grading root surface area of three coniferous trees to L-level in the vertical direction, respectively: **(G)** *P. bungeana*; **(H)** *P. armandii*; **(I)** *P. tabulaeformis*.

The root surface area of the three coniferous trees exhibited a similar pattern across different horizontal stratification levels. Pb-L and Pb-M root surface area exhibited an initial upsurge followed by a decline with expanding horizontal space. Furthermore, the root surface area displayed a gradual reduction upon an increase in soil depth (**Figure 7A**). The root surface area of Pb-N increased first and then decreased with the increase of horizontal direction, and decreased first and then increased with the increase of soil depth. The root surface area of Pa-L first increased and then decreased with increasing horizontal direction (**Figure 7B**). The root surface of Pa-M and Pa-N decreased with increasing horizontal direction. In the vertical direction, the root surface areas of Pa-L, Pa-M and Pa-N were 0-20 cm>20-40 cm>40-60 cm, 0-20 cm>40-60 cm>20-40 cm, 0-20 cm<20-40 cm<40-60 cm respectively. Under different growth conditions, the root surface area of *P. tabulaeformis* first increased and then decreased with increasing horizontal direction (**Figure 7C**). In the vertical direction, Pt-L and Pt-M in the horizontal direction A, compared with 0-20 cm, the root surface areas of 20-40 cm and 40-60 cm decreased by 11.95% and 35.14% respectively 40.51% and 33.75%. In horizontal direction A of Pt-N, the root surface areas of 20-40 cm and 40-60 cm increased by 160.53% and 100.58% respectively compared with 0-20 cm.

With increasing growth grading at the same soil depth, the root surface area of both absorbing roots and transporting roots of the three coniferous trees underwent a substantial decrease, with the surface area of absorbing roots being lower compared to that of transporting roots (**Figures 7D–F**). The ratio of Pb-N root surface area to Pb-L increased with increasing soil depth; The ratio of root surface area to Pb-L of Pb-M increased first and then decreased with increasing root diameter at 0-40 cm, and the ratio of root surface area to Pb-L of Pb-N at 40-60 cm was higher than 0-40 cm (**Figures 7G–I**). As the soil depth increased, there was a rise in the ratio of root surface area to Pa-L in both Pa-M and Pa-N. The ratio of root surface area to Pt-L was higher at 40-60 cm soil depth than at 0-40 cm, and the ratio of root surface area to Pt-L was lower at 0-20 cm soil depth than at 20-60cm.

**Figure 8:**
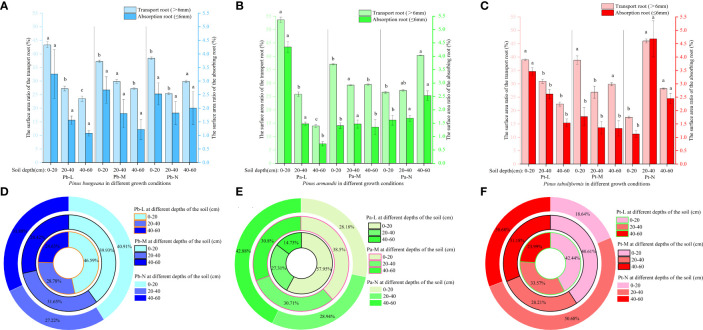
Effect of high soil water content on the proportion of root surface area of three conifers: The proportion distribution of root surface area of graded root: **(A)** *P. bungeana*; **(B)** *P. armandii*; **(C)** *P. tabulaeformis*. The proportion of root surface area in vertical direction: **(D)** *P. bungeana*; **(E)** *P. armandii*; **(F)** *P. tabulaeformis*.

At a depth of 0-20 cm, Pb-L demonstrated a markedly higher proportion of transport root surface area compared to Pb-M and Pb-N (**Figures 8A–C**). In the 40-60 cm soil layer, the proportion of transport root surface area was Pb-N>Pb-M>Pb-L, showing a significant difference. Pa-L exhibited a significantly higher proportion of transport root surface area at a depth of 0-20 cm compared to Pa-M and Pa-N. The proportion of transport root surface area of Pa-N in 40-60 cm soil layer was significantly higher than that of Pa-L and Pa-M. At a 0-20 cm soil depth, Pt-L exhibited a notably higher proportion of absorbing root surface area as compared to Pt-M and Pt-N. The proportion of root surface area of three coniferous trees was different at different soil depths (**Figures 8D–F**). At a depth of 0-20 cm, the proportion of root surface area of 3 kinds of conifers were Pb-L>Pb-N>Pb-M, Pa-L>Pa-M>Pa-N, Pt-L>Pt-M>Pt-N. At the depth of 40-60 cm, the proportion of root surface area of *P. bungeana*, *P. armandii* and *P. tabulaeformis* of N and L grades increased by 29.41%, 191.02% and 27.89%, respectively.

#### 3.4.4 Root volume

**Figure 9:**
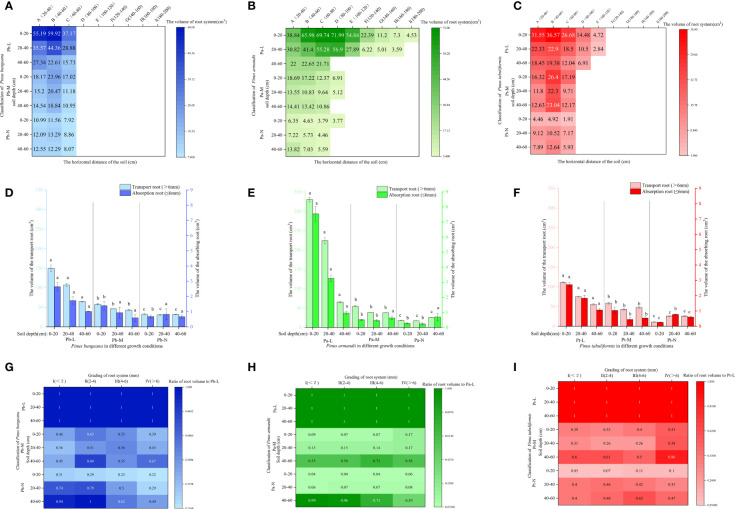
Effect of high soil water content on root system volume of three coniferous trees. Distribution of root volume of three coniferous trees in the horizontal and vertical directions: **(A)** *P. bungeana*; **(B)** *P. armandii*; **(C)** *P. tabulaeformis*. The vertical distribution of the volume of three coniferous trees grading roots: **(D)** *P. bungeana*; **(E)** *P. armandii*; **(F)** *P. tabulaeformis*. The ratio of grading root volume of three coniferous trees to L-level in the vertical direction, respectively: **(G)** *P. bungeana*; **(H)** *P. armandii*; **(I)** *P. tabulaeformis*.

The root volume of Pb-M first increased and then decreased with an increasing horizontal range at different soil depths (**Figure 9A**). The root volumes of Pb-L and Pb-N first increased and then decreased with an increasing horizontal range at 0-40 cm depth and decreased gradually at 40-60 cm depth. A gradual reduction in the root volumes was observed for both Pa-M and Pa-N as the horizontal range distance increased (**Figure 9B**). With increasing soil depth, the root volume of Pa-L showed a downwards trend, while that of Pa-N showed a reverse trend. The root volumes of *P. tabulaeformis* trees first increased and then decreased with increasing horizontal range distances at different soil depths (**Figure 9C**). At the same level, the root volumes of Pt-L, Pt-M, and Pt-N in different soil depths were 0-20 cm>20-40 cm>40-60 cm, 0-20 cm > 40-60 cm > 20-40 cm, and 0-20 cm< 40-60 cm< 20-40 cm.

The volume of the transport roots of the three conifers at the same soil depths decreased significantly with the deterioration of tree growth (**Figures 9D–F**). The ratio of the Pb-M root volume to the Pb-L increased first and then decreased with the increase of root diameter at 0-40 cm (**Figures 9G–I**). The ratio of root volume to Pa-L of Pa-M and Pa-N increased with the increase of soil depth. The ratio of Pt-M root volume to Pt-L was higher than 0-40 cm at a 40-60 cm soil depth.

**Figure 10:**
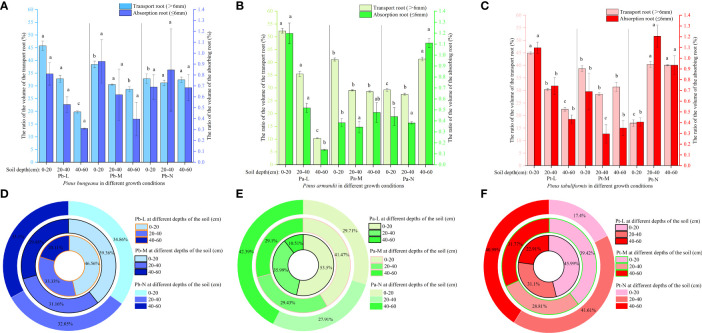
Effect of high soil water content on the proportion of root volume of three conifers. The proportion distribution of root volume of graded root: **(A)** *P. bungeana*; **(B)** *P. armandii*; **(C)** *P. tabulaeformis*. The proportion of root volume in vertical direction: **(D)** *P. bungeana*; **(E)** *P. armandii*; **(F)** *P. tabulaeformis*.

In the 0-20 cm soil layer, the transport root volume proportion of Pb-L was significantly higher than that of Pb-M and Pb-N (**Figures 10A–C**). In the 40-60 cm soil layer, the transport root volume proportion was Pb-N>Pb-M>Pb-L, showing a significant difference. The percentage of Pa-N transport root volume in the 40-60 cm soil layer was significantly higher than that of Pa-L and Pa-M. The volume proportion of transport roots and absorbing roots of Pt-N at 20-40 cm was significantly higher than that of Pt-L and Pt-M. The root volume proportions of *P. armandii*, *P. tabulaeformis* and *P. bungeana* under different growth conditions at 20-40 cm were Pa-L>Pa-M>Pa-N, Pt-N>Pt-L>Pt-M, Pb-L>Pb-N>Pb-M (**Figures 10D–F**).

### 3.5 Changes in root activity of three conifers

**Figure 11:**
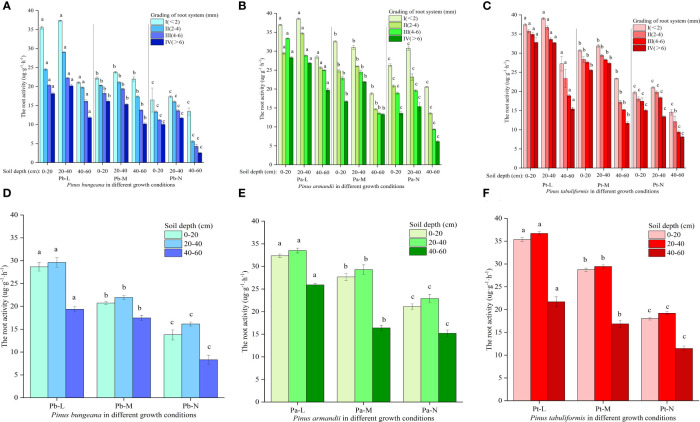
Effect of high soil water content on root activity of three conifers: **(A)** Changes in root activity in hierarchical roots of *P. bungeana*; **(B)** Changes in root activity in hierarchical roots of *P. armandii*; **(C)** Changes in root activity in hierarchical roots of *P. tabulaeformis*; **(D)** The root activity of P. bungeana hierarchical roots changed in the vertical direction of soil; **(E)** The root activity of *P. armandii* hierarchical roots changed in the vertical direction of soil; **(F)** The root activity of *P. tabulaeformis* hierarchical roots changed in the vertical direction of soil.

Root activity is closely related to the absorption levels of water and mineral nutrients by plant roots in the environment, which directly affects the plant’s growth and material accumulation (Qian et al., 2022). Under adverse conditions, root activity is an essential physiological indicator reflecting a plant’s ability to resist stress, and it directly affects the plant’s growth conditions (Liu et al., 2023). The root activity of the three coniferous species increased first and then decreased with increasing soil depth: 20-40 cm>0-20 cm>40-60 cm (**Figure 11**). Root activity demonstrated a decline with not only the deterioration of conifer growth status but also with an increase in root diameter. Compared with Pb-N, the root activity of Pb-L and Pb-M increased significantly by 132.83% and 110.1%, respectively, in 40-60 cm soil layers. (**Figures 11A, D**). At the same root diameter, the root activities of Pb-N, Pb-M and Pb-L showed the following trend: Pb-N<Pb-M<Pb-L. The root activity of *P. armandii* in the vertical soil direction was 40-60 cm<0-20 cm<20-40 cm (**Figures 11B, E**). Compared with Pa-N, the root activity of Pa-L at 0-20 cm, 20-40 cm and 40-60 cm increased by 53.27%, 46.38% and 70.45%, respectively. The root activity of *P. armandii* decreased with increasing root diameter and presented the following trend: Pa-L>Pa-M>Pa-N. The root activity of *P. tabulaeformis* decreased with increasing root diameter and presented a trend of Pt-L>Pt-M>Pt-N (**Figures 11C, F**). The root activity of *P. tabulaeformis* in the vertical direction showed a trend of 20-40 cm > 0-20 cm > 40-60 cm in soil depths and a trend of Pt-L > Pt-M > Pt-N at the same soil depths.

### 3.6 Changes in root respiration of three conifers

**Figure 12:**
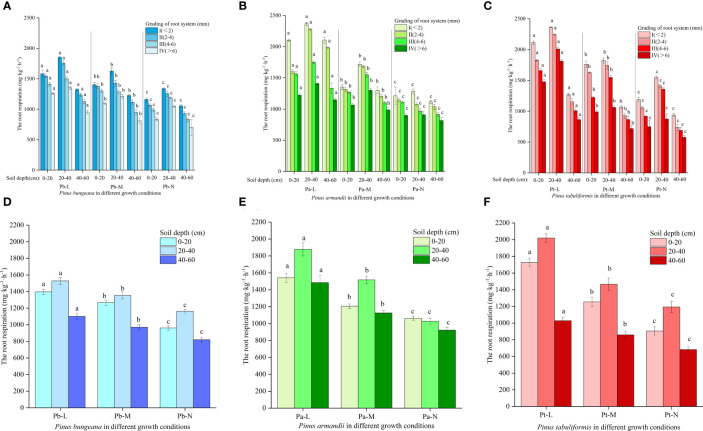
Effect of high soil water content on root respiration of three conifers: **(A)** Changes in root respiration in hierarchical roots of *P. bungeana*; **(B)** Changes in root respiration in hierarchical roots of *P. armandii*; **(C)** Changes in root respiration in hierarchical roots of *P. tabulaeformis*; **(D)** The root respiration of *P. bungeana* hierarchical roots changed in the vertical direction of soil; **(E)** The root respiration of *P. armandii* hierarchical roots changed in the vertical direction of soil; **(F)** The root respiration of *P. tabulaeformis* hierarchical roots changed in the vertical direction of soil.

The root respiration of three coniferous trees gradually decreased with increasing root diameter, and showed the following trend at different soil depths: 20-40 cm > 0-20 cm > 40-60 cm (**Figures 12A–C**). The root respiration rates of the three conifers decreased gradually with increasing growth status: Pb-L>Pb-M>Pb-N, Pa-L>Pa-M>Pa-N, and Pt-L>Pt-M>Pt-N. At the same soil depths, the root respiration of three conifers under different growth conditions were significantly different. The root respiration rates were as follows: Pa-L>Pa-M>Pa-N, Pb-L>Pb-M>Pb-N, and Pt-L>Pt-M>Pt-N (**Figures 12D–F**). With increasing soil depth, the root respiration of the three conifers showed a trend of first rising and then declining: 20-40 cm>0-20 cm>40-60 cm. At a depth of 20-40 cm, there was a significant increase in the root respiration of Pa-L and Pa-M as compared to the other two soil layers. Compared with the other two soil layers, the root respiration rate of Pa-N decreased significantly at 40-60 cm. With increasing soil depth, the root respiration rates of Pt-N and Pt-L decreased by 47.54%, 40.86%, and 33.60%. At a 0-20 cm soil depth, Pb-L and Pb-M increased by 45.21% and 31.66% respectively, compared with Pb-N. At a 40-60 cm soil depth, the Pb-L and Pb-M root respiration rates increased by 34.31% and 18.38%, respectively, compared with Pb-N.

## 4 Discussion

### 4.1 Relationship between soil texture, water content, and aeration at different depths

Soil texture, namely, soil mechanical composition, refers to the relative proportion of soil particles at all levels in the soil and their sand viscosity properties (Kou et al., 2013). Lighter soil has more macroporous structures inside. Therefore, it has high water conductivity and good internal connectivity, which makes the infiltration capacity of soil water stronger (Zhang et al., 2005). Clay is a component of soil colloids and an important material basis for the formation of fertile soil structures. Consequently, clay content directly affects the coordination of many factors such as soil water, fertilizer, gas, and heat (Zhang, 1988). This is consistent with the results of this study. Compared with the surface soil, the 40-60cm soil had higher clay content, lower sand content and higher water content and poor permeability. A high surface temperature in the rainy season may lead to o the significant evaporation of surface soil water (Li et al., 2021). Moreover, the 40-60 cm soil layer was characterized by a predominant clay content in combination with a low sand content, heavy texture, and poor water infiltration capability. These factors could potentially account for the high water content observed in this soil layer (Tan et al., 2019; Wu et al., 2022). When water occupies the pores of the soil, the air in the soil is squeezed out, which may indirectly lead to the lowest permeability coefficient of 40-60cm soil. Soil surface evaporation may be higher in summer when grass plants are abundant. Thus the water consumption of 0-20cm soil on the surface is larger, and its water content is lower than that of 20-40cm soil (Shen, 2021).

Excessive soil moisture content leads to the replacement of air in the soil pores (especially O_2_) with water, resulting in a significantly lower diffusion rate of air in water (one-tenth that in air). Thus, due to the slow diffusion of gas in the soil and the competition between soil microorganisms and crops for residual oxygen in the soil, the O_2_ in the soil is rapidly reduces, and the aerobic respiration of roots is affected (Blom and Voesenek, 1996; Panozzo et al., 2019). The growth and development of peach (Wang et al., 2022), kiwifruit (Yuan et al., 2022) and willow (Yang et al., 2022) are inhibited in anoxic environments. In our experimental conditions, the three coniferous trees were in a high water content environment, which resulted in anoxia in the rhizosphere soil. Under anoxic environment, trees not only change their shape, but also initiate physiological and ecological mechanisms to alleviate the harm caused by stress. In this study, soil permeability decreased with the increase of soil water content. Additionally, Xiong’an New Area has increased rainfall in the rainy season; the groundwater level becomes high, which causes the soil to be in a high water content environment for a long time. Furthermore, the three conifers exhibit robust tolerance to drought while also demanding high levels of root respiration. Thus soil with high water content is harmful to them (Jin and Peng, 2022; Bing, 2021; Zhao et al., 2021).

### 4.2 Effects of high soil water content on the phenotypes of three conifers

In response to external biological or abiotic factors, plants adjust or alter their morphological structures to mitigate potential disaster (Peter and Liina, 2000). The harm of soil high humidity stress to plants is mainly caused by secondary stress caused due to excessive soil water, such as hypoxia or anoxia. In the absence of oxygen, plants undergo modifications in their morphological structures and display indications such as leaf yellowing, drooping, leaf curling, and wilting. In addition, the growth of new leaves and shoots is inhibited and senescence is accelerated (Ploschuk et al., 2018). Over time, the growth rate of plants decelerates, and they may eventually wither and die (Zhang et al., 2019). When exposed to high soil moisture environments, plants exhibit predominant signs, such as lack of leaf development, chlorophyll loss, paleness, curling, decay, and lodging (Zhang, 2019). The mulberry experiment also verified this conclusion (Rao et al., 2021). And the N-level of three conifers showed the phenomena of leaf wilting and withering. High water stress in plants can result in the production of detrimental compounds such as reactive oxygen species (ROS). These compounds can swiftly target the chloroplasts in leaves, ultimately leading to the onset of chlorosis and senescence (Yordanova et al., 2004). Another reason is that the increase in anaerobic respiration and hypoxia will lead to the excessive accumulation of carbon dioxide and toxic compounds, leading to accelerated senescence and abscission (Ou et al., 2011).

### 4.3 Effect of high soil water content on photosynthetic pigments of three coniferous leaves

Under soil water stress, plants undergo intensified chlorophyll degradation, leaf aging, and decreased photosynthetic performance (Ni et al., 2016; Zhang et al., 2022). Chlorophyll is a carrier for plants to absorb, transfer, and convert light energy in photosynthesis (Ning et al., 2021). Carotenoids can not only assist plants in light absorption but also act as antioxidants to remove free radicals generated in photorespiration (Cao et al., 2003). The test results showed that the high soil water content had an impact on the photosynthetic pigments of three coniferous leaves, and the contents of chlorophyll a, chlorophyll b and carotenoids in the leaves showed a downwards trend, which was similar to the previous research results of *Paulownia forunei*, *Camelina sativa* and *cotton* (Liu X, et al., 2020; Zhang et al., 2021; Stasnik et al., 2022). The accumulation of active oxygen in conifers during hypoxic or anoxic conditions is believed to exacerbate the peroxidation of membrane lipids found in chloroplasts. This, in turn, can inflict damage on the chlorophyll membrane system and accelerate the degradation of chlorophyll.

### 4.4 Effect of high soil water content on the root biomass of three conifers

Plant biomass is the most direct indicator of plant growth status and environmental adaptability. Most studies have shown that high soil moisture harms plant growth, and changes in plant biomass are the most direct reflection of adaptation to an environment. On the other hand, plant root systems are considered the earliest and most sensitive sites for injury due to hypoxia (Chen et al., 2015). It has been found that plant root biomass and root shoot ratios will decrease due to hypoxia (Guo et al., 2011). The root growth of alfalfa and soybeans is inhibited in anoxic environments, and the inhibition of root systems is strengthened with an increase in oxygen content in soil (Dong, 2011; Yan, 2013). The same conclusion was reached in this study. The root biomass, in order, of the three coniferous trees was L>M>N. The main reason was that the planting depths of M and N trees level were deep. The water content in the soil with a vertical depth of 40-60 cm was higher than at 0-40 cm, the oxygen content was lower than at 0-40 cm, and the inhibition effect on the root system was greater than at 0-40 cm. The root biomass ratio of *P. bungeana* showed different trends in 0-40 cm and 40-60 cm under three different growth conditions. It may be that the reduction in soil oxygen content caused a stress reaction in *P. bungeana*. The root biomass ratio of *P. tabuliformis* showed different trends in 0-40 cm and 40-60 cm under three different growth conditions. The differences in their growth may be because the oxygen stress in the deep soil was stronger than in the surface soil. A higher proportion of root biomass in deep soil layers corresponds to greater stress levels. This disproportion impedes the complete fulfillment of soil root respiration metabolism, thus diminishing the supply of water and nutrients to the ground.

### 4.5 Effect of high soil water content on root morphology of three coniferous trees

The soil environment as well as the function of vegetation is determined by the spatial distribution features of roots. The distribution properties of roots significantly influence the soil environment and the development of intricate above-ground components. At the same time, they are also affected by soil ecological environmental conditions. They are the link between plants and the environment, and they are especially affected by water and ventilation conditions (Song and Wang, 2007). Differences in the distribution patterns of plant root systems are not only related to biological characteristics but are also influenced by environmental factors such as nutrients, water, and space. These adaptations allow plants to stabilize their own growth and strengthen the absorption of soil nutrients and water, resulting in unique growth forms. Root length, surface area, and volume reflect the occupation of resources by root systems and the plant’s ability to absorb soil nutrients and water (Pan et al., 2022). Root morphology plays a crucial role in the absorption and utilization of water and nutrients by roots (Lynch, 1995), and can respond to changes in soil moisture to some extent (Comas et al., 2013). For example, root length, surface area, and volume are vital indicators directly related to soil moisture (Zeng et al., 2013).

In moist soil environments, roots cannot acquire ample oxygen to carry out aerobic respiration. As directly damaged organs, their morphological development is often significantly affected (Peng et al., 2020). Due to anoxic stress and high soil water content, the photosynthetic performance of the aboveground parts of the plants decreases, which leads to a reduction in the distribution ratio of photosynthate to the root system; the growth of the root system is blocked; the total amount of the root system is reduced, leading to programmed cell death; and, ultimately, the volume, length, and surface area of the root system are significantly reduced (Voesenek et al., 1989; Rubio et al., 1995; Mohd et al., 2010). Ma found that water stress significantly reduced wheat root volume, length, and surface area (Ma et al., 2020). Li’s research on alfalfa showed that water stress significantly inhibited the growth of alfalfa storage roots, and root length, volume, and surface area showed a downward trend (Li et al., 2020). However, some research results have shown that the total root length, surface area, and volume of Daphne grandiflora increased first and then decreased (Long et al., 2022). This study indicated that high soil moisture significantly hampers the growth and development of the root systems of the three conifer species. The root length, surface area, and volume trend of the three conifers was L>M>N. The root distribution range of Pa-L and Pt-L in the horizontal direction was larger than that of Pa-M, Pa-N, Pt-M, and Pt-N, indicating that the high soil water content led to the reduction in root horizontal expansion. In the horizontal direction, the roots of Pb-L, Pb-M, and Pb-N were horizontally distributed to 60-80 cm, and there was no difference in the range of horizontal distribution, indicating that the horizontal distribution range of *P. bungeana* roots is less affected by a high-soil-moisture-content environment. The stunted growth of the three coniferous trees is correlated with a reduction in the surface area, volume, and length of both transport and absorbing roots. Additionally, the proportion of transport roots surpassed that of absorbing roots, potentially stemming from hypoxia-induced damage, a decrease in the proportion of roots, and the decline, as well as demise, of numerous absorbing roots. These factors combine to adversely impact the overall growth and development of plants.

### 4.6 Effect of high soil water content on the root activity of the three conifers

Soil moisture stress primarily affects the root system, with above-ground damage symptoms being a result of secondary injuries. An essential indicator of plant root resistance is the measurement of root activity, offering a comprehensive view of the functions of root systems, including absorption, synthesis, and respiration. Cherry tomatoes (Liu C. et al., 2020) and pepper (Zhang et al., 2009) showed a trend of first increasing and then decreasing root activity in environments with high soil water content. Liu showed that the root activity of sesame seedlings in environments with high soil water content was lower than that of a control, and the root activity gradually decreased with time (Liu et al., 2001). In this experiment, the root activity of the three coniferous trees in the M-level and N-level was significantly lower than that of the experimental group in the L-level, and the root activity in different soil depths showed the following trend: 40-60 cm< 0-20 cm< 20-40 cm. This shows that the soil moisture content at depths of 0-40 cm was lower than at depths of 40-60 cm, resulting in higher soil aeration than at depths of 40-60 cm. Therefore, the root activity of coniferous trees at 0-40 cm was higher than at depths of 40-60 cm. A majority of plants exhibit a decline in root activity under high soil moisture stress, primarily driven by the accumulation of free radicals damaging the tissue cells in a hypoxic soil environment. Subsequently, anaerobic respiration acts as a roadblock, contributing to the inhibition of root activity (He et al., 2022).

### 4.7 Effect of high soil water content on the root respiration rates of the three conifers

High soil moisture content leading to hypoxia stress significantly curbs the aerobic respiration of plants, favoring anaerobic respiration instead (Olorunwa et al., 2022). Chen et al. (2008) believed that the respiration rate of the roots of the two sweet cherry varieties decreased in soil with long term high water content; the anaerobic respiration of the roots increased, the rate of the tricarboxylic acid cycle decreased, and the acetaldehyde and ethanol content increased, resulting in more consumption of respiratory substrates and a decrease in the respiration rate of the plant roots (Wang et al., 2009). In this experiment, the root respiration rates of the three conifers also showed a gradual downward trend. At a soil depth of 0-40 cm, soil water content was lower than at 40-60 cm and aeration was better than at 40-60 cm; thus, the root respiration rate at 40-60 cm was lower.

## 5 Conclusion

High soil moisture negatively impacts the physiological and biochemical functioning of plant roots, which causes a cascade of changes in plant growth and morphology. In the Xiong’an New Area, the deep-planting of certain conifers is leading to an anoxic stress situation brought on by excessive soil moisture, ultimately interfering with the normal growth and development of these conifers. Root growth in *P. tabulaeformis*, *P. bungeana*, and *P. armandii* was inhibited due to anoxic stress, which led to a decrease in root biomass, length, surface area, and volume and showed differences at different soil depths. The soil moisture content was found to be at higher levels in the 40-60cm depth, increasing the severity of anoxic stress. Consequently, conifers with a larger proportion of roots in this layer showed poorer growth. The three conifers exhibited reduced growth in an anoxic environment resulting from elevated soil moisture. The death of absorbing roots due to anoxia has evidently impacted the growth of plants. A downtrend was observed in the root respiration and root activity of the three conifers at growing levels M and N compared to level L. Therefore, while planting these conifers in high-moisture soil areas of China, such as the Xiong’an New Area, other options like shallow planting, terrain elevation or mixing with waterlogging resistant trees demanding considerable water could be considered. In this experiment, root growth was poor in the soil at 40-60cm depth. Therefore, shallow planting and soil topography elevation could be considered to reduce the impact of excessive soil water on the anoxic stress of plant roots. As the moisture content of shallow soil is easily influenced by water absorption by vegetation roots, mixing three types of conifers with waterlogging resistant trees with high water demands will help reduce the anoxic stress brought on by high soil moisture to the roots of conifers (Wang et al., 2012; Xie et al., 2022). Moving ahead, our research will focus on exploring various strategies to mitigate anoxic stress on the three conifers growing in environments with high-content soil moisture.

## Data availability statement

The original contributions presented in the study are included in the article/**Supplementary Material**. Further inquiries can be directed to the corresponding author.

## Author contributions

All authors contributed to the study conception and design. Conceptualization is performed by BL and XR. Writing-original draft is performed by XR and BL. Investigation was performed by SQ. Data curation is performed by YD, CM and BL. Resources is performed by CM, HM and BL. Methodology is performed by CM and BL. Formal analysis was performed by BL, XG, YZ and YD. Funding acquisition, Writing-review & editing were performed by BL and XR. All authors commented on previous versions of the manuscript. All authors contributed to the article and approved the submitted version.
