# Supplementary material for: Study on the causes of growth differences in three conifers after the rainy season in the Xiong’an New Area
Source: Front Plant Sci. 2023 Jul 4;14:1176142. doi: 10.3389/fpls.2023.1176142 (PMC10352786; doi:10.3389/fpls.2023.1176142)
Supplement: Supplementary file 1 [file DataSheet_1.docx]

Supplementary Material

Study on the causes of growth differences in three conifers after the rainy season in the Xiong'an New Area

Xin Ran^1^, Shenqi Qiao^1^, Yu Zhang^1^, Xiaokuan Gao ^2^, Yuewei Du ^1^, Bingxiang Liu ^1,3*^, Changming Ma ^1^ and Hongxiang Mu ^1^

*** Correspondence:** Bingxiang Liu: [proser211@126.com](mailto:proser211@126.com)

# Summary

**Figure S1**. Schematic diagram of root sampling: (a) Sampling top view; (b) Sample the flat view.

**Figure S1**. Schematic diagram of root respiration determination.

**Figure S1A.** Composition of soil particles at different depths

**Figure S1B.** Soil water content and field water capacity

**Figure S1C.** Soil bulk density and porosity

**Figure S1D.** The correlation between soil water content and aeration

**Table S2.** Changes in ground diameter and DBH of three coniferous species

**Table S3.** Changes in photosynthetic pigments of three conifers under different growth states

**Figure S4A-4C.** Effects of high soil water content on the distribution of root biomass in the vertical and horizontal directions of three conifers

**Figure S4D-4F.** Effects of high soil water content on the ratio distribution of root biomass in the vertical and horizontal directions of three conifers

**Figure S4G-4I.** The percentage of root biomass in the vertical direction of soil for three conifers

**Figure S5A-5C.** Effects of high soil water content on the horizontal and vertical distributions of root length in three coniferous species.

**Figure S5D-5F.** Distribution of the length of graded roots in the vertical direction in three coniferous species

**Figure S5G-5I** The ratio of graded root length to grade 1 in the vertical direction in three coniferous species

**Figure S6A-6C** Vertical distribution ratio of graded root length of three conifers

**Figure S6D-6F.** The percentage of root length in the vertical direction of soil for three conifers

**Figure S7A-7C.** Distribution of root surface area in the vertical and horizontal directions of three coniferous species

**Figure S7D-7F.** Distribution of the surface area of graded roots in the vertical direction in three coniferous species

**Figure S7G-7I.** The ratio of graded root surface area to grade 1 in the vertical direction in three coniferous species

**Figure S8A-8C.** Vertical distribution ratio of graded root surface area of three conifers

**Figure S8D-8F.** The percentage of root surface area in the vertical direction of soil for three conifers

**Figure S9A-11C.** Distribution of root volume in the vertical and horizontal directions of three coniferous species

**Figure S9D-9F.** Distribution of the volume of graded roots in the vertical direction in three coniferous species

**Figure S9G-9I.** The ratio of graded root volume to grade 1 in the vertical direction in three coniferous species

**Figure S10A-10C** .Vertical distribution ratio of graded root volume of three conifers

**Figure S10D-10F.** The percentage of root volume in the vertical direction of soil for three conifers

**Figure S11A-11C.** Effects of high soil water content on root activity of three coniferous species with graded roots

**Figure S11D-11F.** Root activity of the three conifer root classes varied in the vertical direction of soil

**Figure S12A-12C.** Effects of high soil water content on root respiration of three coniferous species with graded roots

**Figure S12D-12F.** Root respiration of the three conifer root classes varied in the vertical direction of soil


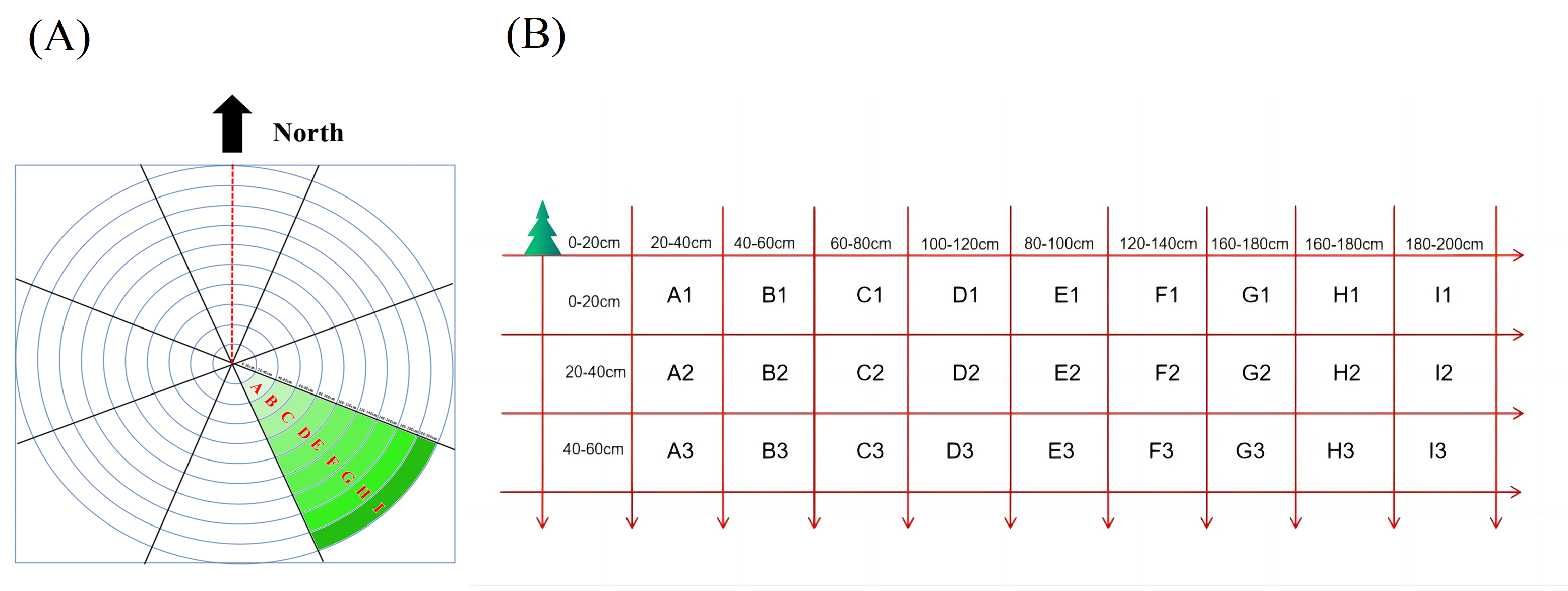


**Figure S1.** Schematic diagram of root sampling: (a) Sampling top view; (b) Sample the flat view.


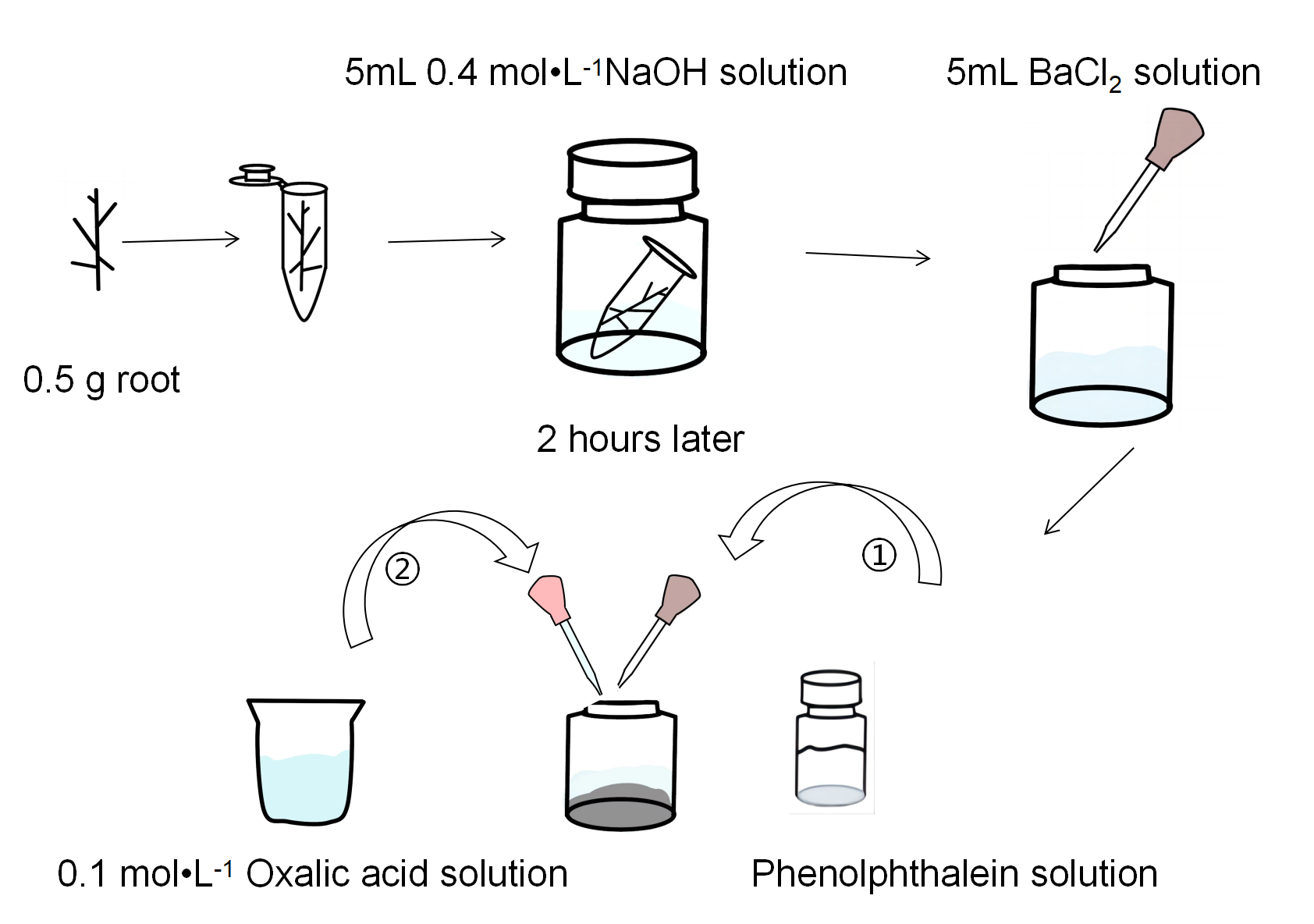


**Figure S2.** Schematic diagram of root respiration determination

**Figure S1A.** Composition of soil particles at different depths(data in Figure 1A)

| [soil depth(cm)](D:\\Program Files (x86)\\Youdao\\Dict\\9.1.2.0\\resultui\\html\\index.html" \l "\\javascript:;" \o "file:///D:\\Program Files (x86)\\Youdao\\Dict\\9.1.2.0\\resultui\\html\\index.html#\\javascript:;) | Particl size composition(%) | | |
| --- | --- | --- | --- |
|  | Clay(＜0.002) | Silt(0.002-0.02) | Sand(0.02-2.00) |
| 0-20 | 5.89±1.72a | 29.88±0.66ab | 64.23±2.34ab |
| 20-40 | 4.15±0.66a | 21.38±6.07b | 74.47±6.62a |
| 40-60 | 7.25±2.20a | 35.97±3.93a | 56.78±6.07b |

**Figure S1B.** Soil water content and field water capacity(data in Figure 1B)

|  | [soil depth(cm)](D:\\Program Files (x86)\\Youdao\\Dict\\9.1.2.0\\resultui\\html\\index.html" \l "\\javascript:;" \o "file:///D:\\Program Files (x86)\\Youdao\\Dict\\9.1.2.0\\resultui\\html\\index.html#\\javascript:;) | | |
| --- | --- | --- | --- |
| Indicator | 0-20 | 20-40 | 40-60 |
| Soil volumetric moisture content(%) | 67.18±15.66b | 75.22±3.23b | 103.01±10.26a |
| Maximum field water capacity(%) | 96.56±3.93a | 93.87±5.44a | 103.33±3.18a |

**Figure S1C** Soil bulk density and porosity(data in Figure 1C)

|  | [soil depth(cm)](D:\\Program Files (x86)\\Youdao\\Dict\\9.1.2.0\\resultui\\html\\index.html" \l "\\javascript:;" \o "file:///D:\\Program Files (x86)\\Youdao\\Dict\\9.1.2.0\\resultui\\html\\index.html#\\javascript:;) | | |
| --- | --- | --- | --- |
| Indicator | 0-20 | 20-40 | 40-60 |
| [Soil bulk density](D:\\Program Files (x86)\\Youdao\\Dict\\9.1.2.0\\resultui\\html\\index.html" \l "\\javascript:;" \o "file:///D:\\Program Files (x86)\\Youdao\\Dict\\9.1.2.0\\resultui\\html\\index.html#\\javascript:;) | 1.2707±0.01308b | 1.1721±0.01423c | 1.3422±0.02086a |
| [Soil porosity](D:\\Program Files (x86)\\Youdao\\Dict\\9.1.2.0\\resultui\\html\\index.html" \l "\\javascript:;" \o "file:///D:\\Program Files (x86)\\Youdao\\Dict\\9.1.2.0\\resultui\\html\\index.html#\\javascript:;) | 52.0501±0.49344b | 55.769±0.53687a | 49.3518±0.78707c |

**FigureS1D** The correlation between soil water content and aeration(data in Figure 1D)

| Water content of 0-20cm soil | Coefficient of air permeability of 0-20cm soil | Water content of 20-40cm soil | Coefficient of air permeability of 20-40cm soil | Water content of 40-60cm soil | Coefficient of air permeability of 40-60cm soil |
| --- | --- | --- | --- | --- | --- |
| 28.74 | 0.09±0.00 | 31.11 | 0.08±0.01 | 33.87 | 0.01±0.001 |
| 28.57 | 0.06±0.00 | 34.87 | 0.09±0.01 | 34.44 | 0.01±0.001 |
| 28.6 | 0.02±0.00 | 30.81 | 0.09±0.00 | 31.74 | 0.01±0.001 |
| 29.06 | 0.02±0.00 | 29.82 | 0.08±0.01 | 20.04 | 0.08±0.002 |
| 30.54 | 0.06±0.01 | 29.5 | 0.08±0.03 | 1.88 | 0.45±0.000 |
| 22.87 | 0.11±0.00 | 20.43 | 0.12±0.02 | 7.96 | 0.23±0.007 |
| 21.7 | 0.11±0.01 | 24.56 | 0.11±0.00 | 28.38 | 0.05±0.002 |
| 23.16 | 0.08±0.01 | 21.75 | 0.14±0.01 | 29.49 | 0.03±0.001 |
| 10.39 | 0.16±0.01 | 16.29 | 0.13±0.01 | 25.66 | 0.05±0.003 |
| 14.92 | 0.14±0.02 | 10.32 | 0.13±0.01 | 17.35 | 0.11±0.003 |
| 13.77 | 0.16±0.01 | 13.68 | 0.18±0.01 | 6.55 | 0.30±0.020 |
| 15.9 | 0.12±0.01 | 3.11 | 0.31±0.01 | 4.33 | 0.38±0.020 |
| 11.08 | 0.18±0.01 | 5.05 | 0.29±0.01 | 3.04 | 0.42±0.024 |
| 11.57 | 0.13±0.01 | 2.75 | 0.26±0.02 | 3.84 | 0.41±0.037 |
| 6.63 | 0.22±0.01 | 3.33 | 0.33±0.03 | 4.47 | 0.34±0.014 |
| 6.05 | 0.27±0.02 | 1.64 | 0.29±0.01 | 2.74 | 0.42±0.043 |
| 4.21 | 0.29±0.01 | 2.05 | 0.24±0.01 | 4.64 | 0.35±0.017 |
| 3.53 | 0.27±0.02 | 1.99 | 0.32±0.00 | 2.87 | 0.43±0.024 |
| 4.25 | 0.35±0.03 | 2.14 | 0.24±0.01 |  |  |
| 4.69 | 0.31±0.01 | 4.32 | 0.42±0.02 |  |  |
| 2.56 | 0.31±0.01 | 2.66 | 0.4±0.02 |  |  |
| 2.4 | 0.36±0.07 | 1.25 | 0.31±0.02 |  |  |
| 3.09 | 0.38±0.03 | 1.58 | 0.27±0.02 |  |  |
| 3.27 | 0.33±0.01 | 1.53 | 0.33±0.01 |  |  |
| 1.85 | 0.34±0.01 |  |  |  |  |
| 0.02 | 0.26±0.01 |  |  |  |  |
| 0.02 | 0.38±0.04 |  |  |  |  |
| 0.02 | 0.36±0.03 |  |  |  |  |
| 0.02 | 0.37±0.00 |  |  |  |  |

**Table.2** Changes in ground diameter and DPb-L of three coniferous species(data in Table 2)

| Serial number | Ground diameter(cm) | Diameter at breast height（cm） | The height of tree(cm) | The range of tree-crown(cm) | Leaf length(cm) | The growth of shoots(cm) |
| --- | --- | --- | --- | --- | --- | --- |
| Pa-L | 10.23±0.12a | 6.6±0.32a | 3.76±0.05a | 3.15±0.03a | 8.76±0.24a | 15.04±1.37a |
| Pa-M | 9.40±0.1b | 4.97±0.34b | 3.23±0.02b | 2.82±0.03b | 7.48±0.18b | 7.51±0.38b |
| Pa-N | 9.20±0.12b | 4.27±0.03c | 3.07±0.04c | 2.41±0.03c | 5.86±0.10c | 4.71±0.80c |
| Pb-L | 10.3±0.3a | 2.73±0.2a | 2.81±0.03a | 2.16±0.02a | 6.63±0.18a | 19.08±2.17a |
| Pb-M | 8.4±0.57b | 2.00±0.21b | 2.18±0.01b | 1.81±0.02b | 5.00±0.05b | 10.28±0.86b |
| Pb-N | 7.57±0.23c | 1.97±0.09c | 1.94±0.06c | 1.66±0.04c | 4.47±0.1c | 4.35±0.32c |
| Pt-L | 9.83±0.22a | 3.87±0.39a | 2.68±0.03a | 2.52±0.02a | 15.17±0.22a | 25.25±1.5a |
| Pt-M | 7.40±0.46b | 3.07±0.26b | 2.48±0.04b | 2.36±0.03b | 8.56±0.49b | 16.42±2.16b |
| Pt-N | 7.07±0.29c | 2.63±0.39c | 2.14±0.02c | 2.12±0.07c | 10.57±0.41c | 9.80±1.03c |

| Serial number | [chlorophyll a(mg/g)](D:\\Program Files (x86)\\Youdao\\Dict\\9.1.2.0\\resultui\\html\\index.html" \l "\\javascript:;" \o "file:///D:\\Program Files (x86)\\Youdao\\Dict\\9.1.2.0\\resultui\\html\\index.html#\\javascript:;) | [chlorophyll b(mg/g)](D:\\Program Files (x86)\\Youdao\\Dict\\9.1.2.0\\resultui\\html\\index.html" \l "\\javascript:;" \o "file:///D:\\Program Files (x86)\\Youdao\\Dict\\9.1.2.0\\resultui\\html\\index.html#\\javascript:;) | [Carotenoid(mg/g)](D:\\Program Files (x86)\\Youdao\\Dict\\9.1.2.0\\resultui\\html\\index.html" \l "\\javascript:;" \o "file:///D:\\Program Files (x86)\\Youdao\\Dict\\9.1.2.0\\resultui\\html\\index.html#\\javascript:;) |
| --- | --- | --- | --- |
| Pa-L | 0.77±0.02a | 0.45±0.03a | 0.22±0.00a |
| Pa-M | 0.54±0.00b | 0.35±0.00b | 0.14±0.00b |
| Pa-N | 0.33±0.03c | 0.34±0.05b | 0.10±0.01c |
| Pb-L | 0.60±0.00a | 0.43±0.01a | 0.13±0.00a |
| Pb-M | 0.49±0.00b | 0.42±0.00b | 0.12±0.00b |
| Pb-N | 0.31±0.00c | 0.40±0.00c | 0.08±0.00c |
| Pt-L | 0.83±0.06a | 0.56±0.02a | 0.20±0.00a |
| Pt-M | 0.39±0.04b | 0.42±0.03b | 0.09±0.01b |
| Pt-N | 0.25±0.00c | 0.43±0.00b | 0.07±0.00c |

**Table.S3** Changes in photosynthetic pigments of three conifers under different growth states(data in Table 3)

**Figure S4A-4C.** Effects of high soil water content on the distribution of root biomass in the vertical and horizontal directions of three conifers(data in Figure 4A-4C)

| Indicator | Serial number | Soil depth (cm) | The horizontal distance of the soil (cm) | | | | | | | | |
| --- | --- | --- | --- | --- | --- | --- | --- | --- | --- | --- | --- |
|  |  |  | A(20-40) | B(40-60) | C(60-80) | D(80-100) | E(100-120) | F(120-140) | G(140-160) | H(160-180) | I(180-200) |
| The biomass of the root(g) | Pb-L | 0-20 | 24.62±0.09a | 26.59±0.52a | 22.62±0.92a |  |  |  |  |  |  |
|  |  | 20-40 | 20.92±0.14a | 21.77±0.29a | 10.48±0.07a |  |  |  |  |  |  |
|  |  | 40-60 | 15.27±0.34a | 18.57±0.13a | 8.42±0.1a |  |  |  |  |  |  |
|  | Pb-M | 0-20 | 14.58±0.21b | 16.16±0.5b | 11.56±0.09b |  |  |  |  |  |  |
|  |  | 20-40 | 10.44±0.05b | 10.57±0.24b | 8.42±0.06b |  |  |  |  |  |  |
|  |  | 40-60 | 8.55±0.26c | 8.51±0.12c | 5.23±0.10b |  |  |  |  |  |  |
|  | Pb-N | 0-20 | 12.03±0.23c | 13.63±0.17c | 8.25±0.09c |  |  |  |  |  |  |
|  |  | 20-40 | 6.35±0.10b | 8.59±0.16c | 4.42±0.11c |  |  |  |  |  |  |
|  |  | 40-60 | 10.31±0.14b | 11.03±0.11b | 4.85±0.23b |  |  |  |  |  |  |
|  | Pa-L | 0-20 | 25.4±1.19a | 25.59±0.14a | 27.86±0.29a | 22.20±0.15 | 20.95±1.08 | 18.09±0.03 | 12.75±0.06 | 10.53±0.06 | 2.097±0.06 |
|  |  | 20-40 | 19.72±0.76a | 16.74±0.07a | 21.82±0.43a | 18.31±0.04 | 11.10±0.03 | 9.26±0.05 | 5.90±0.03 | 3.07±0.02 |  |
|  |  | 40-60 | 15.19±0.49a | 13.16±0.01a | 10.26±0.1a |  |  |  |  |  |  |
|  | Pa-M | 0-20 | 17.32±0.2b | 13.47±0.16b | 10.60±0.26b | 6.39±0.08 |  |  |  |  |  |
|  |  | 20-40 | 10.63±0.13b | 5.57±0.11b | 3.30±0.06b | 2.1±0.05 |  |  |  |  |  |
|  |  | 40-60 | 10.63±0.13b | 8.42±0.17b | 6.68±0.17b |  |  |  |  |  |  |
|  | Pa-N | 0-20 | 6.47±0.08c | 4.14±0.06c | 0.76±0.03c | 0.55±0.05 |  |  |  |  |  |
|  |  | 20-40 | 7.27±0.03c | 4.56±0.20c | 1.36±0.02c |  |  |  |  |  |  |
|  |  | 40-60 | 9.32±0.03c | 6.13±0.04c | 2.13±0.03c |  |  |  |  |  |  |
|  | Pt-L | 0-20 | 19.18±0.06a | 21.74±0.09a | 17.14±0.73a | 11.64±0.31 | 9.46±0.05 |  |  |  |  |
|  |  | 20-40 | 16.59±0.17a | 19.68±0.08a | 12.85±0.07a | 8.16±0.03 | 6.34±0.08 |  |  |  |  |
|  |  | 40-60 | 14.29±0.10a | 10.37±0.10a | 8.5±0.09a | 6.48±0.04 |  |  |  |  |  |
|  | Pt-M | 0-20 | 14.13±0.15b | 17.11±0.02b | 9.6±0.08b |  |  |  |  |  |  |
|  |  | 20-40 | 7.18±0.11c | 8.42±0.12c | 5.93±0.0.03b |  |  |  |  |  |  |
|  |  | 40-60 | 10.05±0.08b | 10.32±0.04a | 6.36±0.13b |  |  |  |  |  |  |
|  | Pt-N | 0-20 | 8.63±0.04c | 5.34±0.12c | 3.2±0.13c |  |  |  |  |  |  |
|  |  | 20-40 | 11.29±0.10019b | 10.08±0.03b | 4.25±0.12c |  |  |  |  |  |  |
|  |  | 40-60 | 10.11±0.04b | 8.44±0.21b | 3.83±0.07c |  |  |  |  |  |  |

**Figure S4D-4F.** Effects of high soil water content on the ratio distribution of root biomass in the vertical and horizontal directions of three conifers(data in Figure 4D-4F)

| Indicator | Serial number | Soil depth (cm) | The horizontal distance of the soil (cm) | | | | | | | | |
| --- | --- | --- | --- | --- | --- | --- | --- | --- | --- | --- | --- |
|  |  |  | A(20-40) | B(40-60) | C(60-80) | D(80-100) | E(100-120) | F(120-140) | G(140-160) | H(160-180) | I(180-200) |
| The percentage of root biomass(%) | Pb-L | 0-20 | 0.14±0.002b | 0.16±0.004b | 0.13±0.004a |  |  |  |  |  |  |
|  |  | 20-40 | 0.12±0.002a | 0.13±0.002a | 0.06±0.001b |  |  |  |  |  |  |
|  |  | 40-60 | 0.09±0.003b | 0.11±0.002b | 0.05±0.001b |  |  |  |  |  |  |
|  | Pb-M | 0-20 | 0.16±0.002a | 0.17±0.005a | 0.12±0b |  |  |  |  |  |  |
|  |  | 20-40 | 0.11±0.001b | 0.11±0.002b | 0.09±0.001a |  |  |  |  |  |  |
|  |  | 40-60 | 0.09±0.003b | 0.09±0.001c | 0.06±0.001a |  |  |  |  |  |  |
|  | Pb-N | 0-20 | 0.15±0.003ab | 0.17±0.002a | 0.1±0.001c |  |  |  |  |  |  |
|  |  | 20-40 | 0.08±0.002c | 0.11±0.002b | 0.06±0.001c |  |  |  |  |  |  |
|  |  | 40-60 | 0.13±0.002a | 0.14±0.001a | 0.06±0.003a |  |  |  |  |  |  |
|  | Pa-L | 0-20 | 0.08±0.004c | 0.08±0.001c | 0.09±0.001b | 0.07±0.001a | 0.07±0.00186 | 0.06±0.00033 | 0.04±0.0004 | 0.034±0.0001 | 0.007±0.0001 |
|  |  | 20-40 | 0.06±0.003c | 0.05±0.001b | 0.07±0.001a | 0.06±0.00 | 0.04±0.00021 | 0.03±0.00001 | 0.019±0.00 | 0.01±0.0001 |  |
|  |  | 40-60 | 0.05±0.001c | 0.04±0c | 0.03±0c |  |  |  |  |  |  |
|  | Pa-M | 0-20 | 0.18±0.002a | 0.14±0.002a | 0.11±0.002a | 0.06±0.00b |  |  |  |  |  |
|  |  | 20-40 | 0.11±0.001b | 0.06±0.001b | 0.03±0.001b | 0.02±0.00 |  |  |  |  |  |
|  |  | 40-60 | 0.15±0.002b | 0.09±0.002b | 0.07±0.002a |  |  |  |  |  |  |
|  | Pa-N | 0-20 | 0.15±0.002b | 0.1±0.002b | 0.02±0.001c | 0.01±0.001c |  |  |  |  |  |
|  |  | 20-40 | 0.17±0a | 0.11±0.005a | 0.03±0.001b |  |  |  |  |  |  |
|  |  | 40-60 | 0.22±0.001a | 0.14±0.001a | 0.05±0.001b |  |  |  |  |  |  |
|  | Pt-L | 0-20 | 0.11±0c | 0.12±0b | 0.1±0.001b | 0.07±0.002 | 0.05±0.00033 |  |  |  |  |
|  |  | 20-40 | 0.09±0.001b | 0.11±0b | 0.07±0a | 0.04±0.00 | 0.03±0.00033 |  |  |  |  |
|  |  | 40-60 | 0.08±0.001c | 0.06±0.001c | 0.05±0.001c | 0.04±0.00033 |  |  |  |  |  |
|  | Pt-M | 0-20 | 0.15±0.004a | 0.19±0.005a | 0.11±0.004a |  |  |  |  |  |  |
|  |  | 20-40 | 0.08±0.003c | 0.09±0.003c | 0.07±0.002b |  |  |  |  |  |  |
|  |  | 40-60 | 0.11±0.002b | 0.11±0.003b | 0.07±0.001a |  |  |  |  |  |  |
|  | Pt-N | 0-20 | 0.13±0.001b | 0.08±0.003c | 0.05±0.001c |  |  |  |  |  |  |
|  |  | 20-40 | 0.17±0.003a | 0.15±0.001a | 0.06±0.002b |  |  |  |  |  |  |
|  |  | 40-60 | 0.15±0.002a | 0.13±0.003a | 0.06±0.001b |  |  |  |  |  |  |

F**igure S4G-4I.** The percentage of root biomass in the vertical direction of soil for three conifers(data in Figure 4G-4I)

| Indicator | Serial number | Soil depth (cm) | | |
| --- | --- | --- | --- | --- |
|  |  | 0-20 | 20-40 | 40-60 |
| The percentage of root biomass(%) | Pb-L | 44.33±0.88a | 31.01±0.41a | 24.66±0.47b |
|  | Pb-M | 44.99±0.63a | 31.3±0.26a | 23.72±0.4b |
|  | Pb-N | 42.67±0.47a | 24.37±0.45b | 32.96±0.37a |
|  | Pa-L | 53.32±0.35a | 34.21±0.16a | 12.47±0.19c |
|  | Pa-M | 48.37±0.32b | 21.86±0.11c | 29.77±0.4b |
|  | Pa-N | 27.93±0.46c | 30.89±0.5b | 39.54±1.57a |
|  | Pt-L | 43.54±0.65b | 34.77±0.18b | 21.69±0.14c |
|  | Pt-M | 47.15±1.24a | 23.59±0.71c | 29.26±0.54b |
|  | Pt-N | 27.13±0.87c | 38.9±0.63a | 33.98±0.3a |

**FigureS5A-5C.** Effects of high soil water content on the horizontal and vertical distributions of root length in three coniferous species.(data in Figure 5A-5C)

| Indicator | Serial number | Soil depth (cm) | The horizontal distance of the soil (cm) | | | | | | | | |
| --- | --- | --- | --- | --- | --- | --- | --- | --- | --- | --- | --- |
|  |  |  | A(20-40) | B(40-60) | C(60-80) | D(80-100) | E(100-120) | F(120-140) | G(140-160) | H(160-180) | I(180-200) |
| The length of the root(cm) | Pt-L | 0-20 | 1662.31±4.52a | 1727.18±6.4a | 1442.27±13.03a | 829.86±21.69 | 259.56±9.27 |  |  |  |  |
|  |  | 20-40 | 1185.72±41.38a | 1502.84±38.68a | 1074.7±65.86a | 626.13±32.98 | 128.27±3.76 |  |  |  |  |
|  |  | 40-60 | 858.78±8.17a | 1206.22±48.49a | 678.1±6.29a | 369.34±20.69 |  |  |  |  |  |
|  | Pt-M | 0-20 | 851.76±2.06b | 1394.55±1.31b | 826.81±3.02b |  |  |  |  |  |  |
|  |  | 20-40 | 382.1±1.24c | 863±2.27c | 327.52±1.32b |  |  |  |  |  |  |
|  |  | 40-60 | 447.4±2.25b | 1179.07±4.74a | 464.85±0.44b |  |  |  |  |  |  |
|  | Pt-N | 0-20 | 289.53±4.93c | 415.47±6.39c | 76.93±1.83c |  |  |  |  |  |  |
|  |  | 20-40 | 616.27±3.11b | 940.64±3.74b | 245.97±6.55b |  |  |  |  |  |  |
|  |  | 40-60 | 353.6±1.89c | 562.38±3.74b | 129.18±5.96b |  |  |  |  |  |  |
|  | Pa-L | 0-20 | 2248.32±68a | 2664.18±81.14a | 4471.37±186.43a | 4153.96±29.32a | 2677.23±56.75 | 930.99±20.14 | 536.25±20.74 | 247.55±20.45 | 180.95±19.14 |
|  |  | 20-40 | 1351.64±21.8a | 2174.21±49.38a | 2662.27±18.31a | 570.61±75.38 | 334.20±17.34 | 315.38±5.13 | 241.15±18.9 | 133.54±14.34 |  |
|  |  | 40-60 | 893.54±39.85a | 1255.43±42.23a | 1706.82±55.01a |  |  |  |  |  |  |
|  | Pa-M | 0-20 | 768.42±3.42b | 586.89±3.48b | 540.78±4.17b | 158.75±1.04b |  |  |  |  |  |
|  |  | 20-40 | 644.24±2.62b | 490.71±4.08b | 444.61±3.91b | 111.77±2.36 |  |  |  |  |  |
|  |  | 40-60 | 719.83±3.86b | 540.55±3.84b | 465.51±1.19b |  |  |  |  |  |  |
|  | Pa-N | 0-20 | 359.2±12.41c | 190.48±3.27c | 156.88±3.27c | 137.21±1.99b |  |  |  |  |  |
|  |  | 20-40 | 563.43±4.53c | 291.76±7.4c | 212.97±1.43c |  |  |  |  |  |  |
|  |  | 40-60 | 768.95±9.61b | 222.73±1.4c | 171.76±5.95c | . |  |  |  |  |  |
|  | Pb-L | 0-20 | 2440.45±2.28a | 2543.52±4.54a | 2304.82±1.61a |  |  |  |  |  |  |
|  |  | 20-40 | 1458.75±1.02a | 1673.47±3.45a | 1301.63±4.34a |  |  |  |  |  |  |
|  |  | 40-60 | 1187.89±4.93a | 1482.4±2.21a | 1279.01±1.95a |  |  |  |  |  |  |
|  | Pb-M | 0-20 | 1135.5±3.22b | 1692.95±5.24b | 1146.73±2.16b |  |  |  |  |  |  |
|  |  | 20-40 | 885.13±2.38b | 1372.92±1.12b | 646.62±1.58b |  |  |  |  |  |  |
|  |  | 40-60 | 785.97±3.91b | 1112.99±4.01b | 533.5±4.85b |  |  |  |  |  |  |
|  | Pb-N | 0-20 | 746.07±4.19c | 933.81±2.33c | 585.78±3.5c |  |  |  |  |  |  |
|  |  | 20-40 | 554.19±2.98c | 635.63±2.86c | 405.32±2c |  |  |  |  |  |  |
|  |  | 40-60 | 578.37±3.68c | 647.03±3.13c | 468.8±2.49c |  |  |  |  |  |  |

**FigureS5D-5F.** Distribution of the length of graded roots in the vertical direction in three coniferous species(data in Figure 5D-5F)

| Indicator | Serial number | Soil depth (cm) | Transport root (＞6mm) | Absorption root(≤6mm) |
| --- | --- | --- | --- | --- |
| The length of the root(cm) | Pa-L | 0-20 | 12944.95±280.05a | 4865.56±162.99a |
|  |  | 20-40 | 6136.94±72.06a | 1646.07±71.3a |
|  |  | 40-60 | 3036.41±81.46a | 819.38±79.14a |
|  | Pa-M | 0-20 | 1720.96±19.75b | 333.88±19.58b |
|  |  | 20-40 | 1411.29±11.81b | 280.04±19.08b |
|  |  | 40-60 | 1409.34±36.79b | 316.55±31.15b |
|  | Pa-N | 0-20 | 667.63±28.96c | 176.14±27.08b |
|  |  | 20-40 | 843.5±5.59c | 224.67±16.55b |
|  |  | 40-60 | 890.48±15.85c | 272.95±12.22b |
|  | Pt-L | 0-20 | 4114.1±63.74a | 1807.07±61.2a |
|  |  | 20-40 | 3232.65±64.57a | 1285.01±87.4a |
|  |  | 40-60 | 2363.97±35.53a | 748.47±81.44a |
|  | Pt-M | 0-20 | 2394.23±44.28b | 678.89±45.5b |
|  |  | 20-40 | 1326.39±23.85b | 246.23±27.25b |
|  |  | 40-60 | 1675.26±36.85b | 416.07±37.54b |
|  | Pt-N | 0-20 | 589.6±8.14c | 192.33±9.64c |
|  |  | 20-40 | 1235.13±105.29b | 567.74±106.38c |
|  |  | 40-60 | 794.88±3.04c | 250.28±2.8b |
|  | Pb-L | 0-20 | 6279.58±396.25a | 1788.76±382.21a |
|  |  | 20-40 | 3474.52±69.18s | 957.24±75.47a |
|  |  | 40-60 | 3342.13±39.14a | 607.17±34.32a |
|  | Pb-M | 0-20 | 3153.65±112.56b | 821.54±109.86b |
|  |  | 20-40 | 2367.11±109.57b | 535.68±109.7b |
|  |  | 40-60 | 2065.87±64.41b | 366.6±65.38b |
|  | Pb-N | 0-20 | 1795.96±32.64c | 469.7±33.75b |
|  |  | 20-40 | 1250.74±55.83c | 344.4±55.83b |
|  |  | 40-60 | 1366.12±59.34c | 328.07±62.21b |

**Figure S5G-5I.** The ratio of graded root length to grade 1 in the vertical direction in three coniferous species(data in Figure 5G-5I)

| Indicator | Serial number | Soil depth (cm) | Grading of root system(mm) | | | |
| --- | --- | --- | --- | --- | --- | --- |
|  |  |  | I(＜2) | Ⅱ(2-4) | Ⅲ(4-6) | Ⅳ(＞6) |
| The ratio of graded root length area to grade 1 | Pb-L | 0-20 | 1±0a | 1±0a | 1±0a | 1±0a |
|  |  | 20-40 | 1±0a | 1±0a | 1±0a | 1±0a |
|  |  | 40-60 | 1±0a | 1±0a | 1±0a | 1±0a |
|  | Pb-M | 0-20 | 0.38±0.06b | 0.51±0.14b | 0.55±0.16b | 0.5±0.01b |
|  |  | 20-40 | 0.52±0.16b | 0.55±0.13b | 0.58±0.13b | 0.68±0.03b |
|  |  | 40-60 | 0.49±0.1b | 0.86±0.15a | 0.52±0.12b | 0.62±0.02b |
|  | Pb-N | 0-20 | 0.3±0.06b | 0.28±0.07b | 0.29±0.06b | 0.29±0.01c |
|  |  | 20-40 | 0.52±0.02b | 0.4±0.08b | 0.33±0.06b | 0.36±0.02c |
|  |  | 40-60 | 0.75±0.09ab | 0.81±0.22a | 0.41±0.02b | 0.41±0.01c |
|  | Pa-L | 0-20 | 1±0a | 1±0a | 1±0a | 1±0a |
|  |  | 20-40 | 1±0a | 1±0a | 1±0a | 1±0a |
|  |  | 40-60 | 1±0a | 1±0a | 1±0a | 1±0a |
|  | Pa-M | 0-20 | 0.09±0b | 0.07±0.01b | 0.07±0.01b | 0.13±0b |
|  |  | 20-40 | 0.18±0.02b | 0.18±0.01b | 0.17±0.02b | 0.23±0b |
|  |  | 40-60 | 0.33±0.02b | 0.35±0.04b | 0.44±0.08b | 0.47±0.02b |
|  | Pa-N | 0-20 | 0.04±0.01c | 0.04±0.01c | 0.03±0c | 0.05±0c |
|  |  | 20-40 | 0.12±0.01c | 0.14±0.02b | 0.14±0.01b | 0.14±0c |
|  |  | 40-60 | 0.34±0.06b | 0.36±0.01b | 0.33±0.04b | 0.29±0.01c |
|  | Pt-L | 0-20 | 1±0a | 1±0a | 1±0a | 1±0a |
|  |  | 20-40 | 1±0a | 1±0a | 1±0a | 1±0a |
|  |  | 40-60 | 1±0a | 1±0a | 1±0a | 1±0a |
|  | Pt-M | 0-20 | 0.45±0.09b | 0.37±0.04b | 0.37±0.01b | 0.58±0.02b |
|  |  | 20-40 | 0.17±0.01b | 0.19±0.02b | 0.2±0.03c | 0.41±0.01b |
|  |  | 40-60 | 0.67±0.16b | 0.62±0.12b | 0.54±0.09b | 0.71±0.01b |
|  | Pt-N | 0-20 | 0.08±0.01c | 0.09±0.01c | 0.13±0c | 0.14±0c |
|  |  | 20-40 | 0.39±0.14b | 0.47±0.14b | 0.43±0.04b | 0.38±0.03b |
|  |  | 40-60 | 0.21±0.02b | 0.32±0.03c | 0.38±0.04b | 0.34±0.01c |

**Figure S6A-6C.** Vertical distribution ratio of graded root length of three conifers(data in Figure 6A-6C)

| Indicator | Serial number | Soil depth (cm) | The ratio of the transport root(＞6mm)(%) | The ratio of the absorption root(≤6mm)(%) |
| --- | --- | --- | --- | --- |
| The ratio of the length of the root(%) | Pa-L | 0-20 | 43.96±0.99a | 16.52±0.53a |
|  |  | 20-40 | 20.84±0.33c | 5.59±0.22b |
|  |  | 40-60 | 10.31±0.24a | 2.78±0.27a |
|  | Pa-M | 0-20 | 31.45±0.39b | 6.1±0.35b |
|  |  | 20-40 | 25.79±0.24b | 5.12±0.34b |
|  |  | 40-60 | 25.76±0.7b | 5.78±0.56b |
|  | Pa-N | 0-20 | 21.71±0.89c | 5.72±0.86b |
|  |  | 20-40 | 27.44±0.39a | 7.3±0.48a |
|  |  | 40-60 | 28.96±0.55c | 8.87±0.36c |
|  | Pt-L | 0-20 | 30.37±0.67b | 13.33±0.36a |
|  |  | 20-40 | 23.86±0.64b | 9.48±0.58ab |
|  |  | 40-60 | 17.45±0.27c | 5.52±0.56a |
|  | Pt-M | 0-20 | 35.54±0.7a | 10.08±0.66b |
|  |  | 20-40 | 19.69±0.36b | 3.65±0.4b |
|  |  | 40-60 | 24.87±0.54a | 6.18±0.56a |
|  | Pt-N | 0-20 | 16.24±0.2c | 5.3±0.27c |
|  |  | 20-40 | 34.04±2.95a | 15.63±2.9a |
|  |  | 40-60 | 21.9±0.05b | 6.89±0.08a |
|  | Pb-L | 0-20 | 38.12±0.6a | 10.71±1.74a |
|  |  | 20-40 | 21.18±0.62b | 5.88±0.69a |
|  |  | 40-60 | 20.39±0.85b | 3.71±0.32a |
|  | Pb-M | 0-20 | 33.87±1.21b | 8.82±1.18a |
|  |  | 20-40 | 25.42±1.16a | 5.75±1.18a |
|  |  | 40-60 | 22.18±0.69ab | 3.94±0.7a |
|  | Pb-N | 0-20 | 32.33±0.62b | 8.45±0.6a |
|  |  | 20-40 | 22.52±1.03ab | 6.2±1a |
|  |  | 40-60 | 24.6±1.09a | 5.9±1.11a |

**Figure S6D-6F.** The percentage of root length in the vertical direction of soil for three conifers(data in Figure 6D-6F)

| Indicator | Serial number |  | Soil depth (cm) |  |
| --- | --- | --- | --- | --- |
|  |  | 0-20 | 20-40 | 40-60 |
| The ratio of the length of the root(%) | Pa-L | 60.48±0.47a | 26.43±0.15c | 13.09±0.4c |
|  | Pa-M | 37.55±0.15b | 30.91±0.12b | 31.54±0.17b |
|  | Pa-N | 27.43±0.17c | 34.73±0.19a | 37.83±0.21a |
|  | Pt-L | 43.7±0.32b | 33.34±0.19b | 22.96±0.4c |
|  | Pt-M | 45.62±0.04a | 23.34±0.04c | 31.04±0.04a |
|  | Pt-N | 21.54±0.08c | 49.67±0.17a | 28.79±0.12b |
|  | Pb-L | 48.83±2.32a | 27.07±1.25b | 24.11±1.07b |
|  | Pb-M | 42.69±0.04b | 31.19±0.03a | 26.12±0.01b |
|  | Pb-N | 40.79±0.04b | 28.72±0.13ab | 30.5±0.11a |

**Figure S7A-7C.** Distribution of root surface area in the vertical and horizontal directions of three coniferous species(data in Figure 7A-7C)

| Indicator | Serial number | Soil depth (cm) | The horizontal distance of the soil (cm) | | | | | | | | |
| --- | --- | --- | --- | --- | --- | --- | --- | --- | --- | --- | --- |
|  |  |  | A(20-40) | B(40-60) | C(60-80) | D(80-100) | E(100-120) | F(120-140) | G(140-160) | H(160-180) | I(180-200) |
| The surface area of the root(cm^2^) | Pt-L | 0-20 | 672.48±5.54a | 776.71±6.86b | 684.08±29.35a | 383.84±19.03 | 123.21±13.56 |  |  |  |  |
|  |  | 20-40 | 592.07±3.43a | 650.29±36.16a | 508.32±40.56a | 271.29±15.49 | 67.01±5.17 |  |  |  |  |
|  |  | 40-60 | 436.11±32.5a | 559.1±1.85a | 319.33±3.3a | 178.03±23.01 |  |  |  |  |  |
|  | Pt-M | 0-20 | 394.38±3.56b | 818.35±1.69a | 401.48±26.66b |  |  |  |  |  |  |
|  |  | 20-40 | 234.61±2.56b | 535.94±3.33b | 70.1±12.23b |  |  |  |  |  |  |
|  |  | 40-60 | 261.29±4.28b | 617.66±2.44b | 319.45±0.68a |  |  |  |  |  |  |
|  | Pt-N | 0-20 | 129.59±1.86c | 141.25±2.32c | 45.96±4.88c |  |  |  |  |  |  |
|  |  | 20-40 | 337.64±3.29c | 395.38±5.98c | 128.3±2.13b |  |  |  |  |  |  |
|  |  | 40-60 | 259.94±1.67b | 189.27±4.9c | 72.29±1.91b |  |  |  |  |  |  |
|  | Pa-L | 0-20 | 1030.15±16.33a | 1449.16±101.58a | 1952.19±16.82a | 1794.34±97.67a | 1345.94±92.1 | 496.3±24.59 | 271.45±30.8 | 150.64±12.73 | 100.45±7.40 |
|  |  | 20-40 | 715.89±32.13a | 1056.36±88.08a | 1341.9±68.15a | 298.87±42.97 | 191.70±27.44 | 156.61±8.11 | 122.01±5.60 | 77.51±6.76 |  |
|  |  | 40-60 | 496.94±24.03a | 592.51±12.25a | 1047.15±74.48a |  |  |  |  |  |  |
|  | Pa-M | 0-20 | 423.51±2.7b | 357.12±2.16b | 307.41±2.36b | 80.53±5.78b |  |  |  |  |  |
|  |  | 20-40 | 338.81±2.18b | 276.06±3.05b | 246.64±2.94b | 70.50±3.45 |  |  |  |  |  |
|  |  | 40-60 | 385.16±3.82b | 293.47±2.02b | 256.22±2.4b |  |  |  |  |  |  |
|  | Pa-N | 0-20 | 168.88±1.78c | 101.3±3.53c | 77.26±1.48c | 64.24±1.51b |  |  |  |  |  |
|  |  | 20-40 | 206.76±2.62c | 132.32±2.99b | 84.5±1.09c |  |  |  |  |  |  |
|  |  | 40-60 | 364.83±1.79b | 158.15±1.07c | 104.5±1.59c | . |  |  |  |  |  |
|  | Pb-L | 0-20 | 1181.81±11.1a | 1203.18±12.92a | 1020.62±4.43a |  |  |  |  |  |  |
|  |  | 20-40 | 762.44±7.94a | 821.15±4.1a | 686.71±6.33a |  |  |  |  |  |  |
|  |  | 40-60 | 657.85±2.83a | 699.11±5.41a | 585.98±3.31a |  |  |  |  |  |  |
|  | Pb-M | 0-20 | 502.24±5.29b | 725.66±1.77b | 498.34±1.47b |  |  |  |  |  |  |
|  |  | 20-40 | 424.98±2.67b | 607.33±3.54b | 335.95±2.53b |  |  |  |  |  |  |
|  |  | 40-60 | 382.96±4.81b | 580.36±5.22b | 265.53±3.32b |  |  |  |  |  |  |
|  | Pb-N | 0-20 | 358.61±3.85c | 387.49±3.32c | 245.56±2.23c |  |  |  |  |  |  |
|  |  | 20-40 | 219.88±1.05c | 254.7±1.42c | 185.16±1.25c |  |  |  |  |  |  |
|  |  | 40-60 | 276±3.35c | 287.6±1.14c | 209.12±2.66c |  |  |  |  |  |  |

**Figure S7D-7F.** Distribution of the surface area of graded roots in the vertical direction in three coniferous species(data in Figure 7D-7F)

| Indicator | Serial number | Soil depth(cm) | Transport root (＞6mm) | Absorption root(≤6mm) |
| --- | --- | --- | --- | --- |
| The surface area of the root(cm^2^) | Pa-L | 0-20 | 7781.18±244.51a | 629.77±21.27a |
|  |  | 20-40 | 3747.06±118.24a | 213.79±7.59a |
|  |  | 40-60 | 2030.58±64.19a | 106.02±11.01a |
|  | Pa-M | 0-20 | 1125.86±10.21b | 42.7±3.16b |
|  |  | 20-40 | 887.42±4.71b | 44.6±4.47b |
|  |  | 40-60 | 893.77±10.53b | 41.08±8.72b |
|  | Pa-N | 0-20 | 388.62±4.86c | 23.65±2.76b |
|  |  | 20-40 | 398.93±5.35c | 24.65±1.72c |
|  |  | 40-60 | 590.45±1.75c | 37.03±2.56b |
|  | Pt-L | 0-20 | 2425±26.53a | 215.32±7.81a |
|  |  | 20-40 | 1926.24±55.69a | 162.75±10.17a |
|  |  | 40-60 | 1397.02±45.24a | 95.55±8.77a |
|  | Pt-M | 0-20 | 1272.34±141.65b | 59.25±15.38b |
|  |  | 20-40 | 864.79±17.98b | 43.38±5.44c |
|  |  | 40-60 | 974.69±84.19b | 43.58±9.6b |
|  | Pt-N | 0-20 | 297.7±7.85c | 19.1±2.17c |
|  |  | 20-40 | 781.6±7.58b | 79.71±12.04b |
|  |  | 40-60 | 479.93±6.14c | 41.57±3.07b |
|  | Pb-L | 0-20 | 3434.27±216.8a | 263.13±83.6a |
|  |  | 20-40 | 2147.64±8.82a | 122.66±8.31a |
|  |  | 40-60 | 1857.52±15.05a | 85.42±7.85a |
|  | Pb-M | 0-20 | 1610.7±20.15b | 115.54±20.994b |
|  |  | 20-40 | 1290.19±28.78b | 78.07±22.39ab |
|  |  | 40-60 | 1176.13±14.08b | 52.72±15.75a |
|  | Pb-N | 0-20 | 930.46±13.96c | 61.2±9.8b |
|  |  | 20-40 | 615.57±9.43c | 44.17±10.15b |
|  |  | 40-60 | 724.16±11.9c | 48.56±14.55a |

**Figure7G-7I.** The ratio of graded root surface area to grade 1 in the vertical direction in three coniferous species(data in Figure 7G-7I)

| Indicator | Serial number | Soil depth(cm) | Grading of root system(mm) | | | |
| --- | --- | --- | --- | --- | --- | --- |
|  |  |  | I(＜2) | Ⅱ(2-4) | Ⅲ(4-6) | Ⅳ(＞6) |
| The ratio of graded root surface area to grade 1 | Pb-L | 0-20 | 1±0a | 1±0a | 1±0a | 1±0a |
|  |  | 20-40 | 1±0a | 1±0a | 1±0a | 1±0a |
|  |  | 40-60 | 1±0a | 1±0a | 1±0a | 1±0a |
|  | Pb-M | 0-20 | 0.45±0.06b | 0.54±0.13b | 0.52±0.2b | 0.47±0.03b |
|  |  | 20-40 | 0.47±0.13b | 0.63±0.24b | 0.68±0.24ab | 0.6±0.02b |
|  |  | 40-60 | 0.53±0.12a | 0.87±0.19a | 0.58±0.2ab | 0.63±0.01b |
|  | Pb-N | 0-20 | 0.36±0.09b | 0.34±0.12b | 0.28±0.09b | 0.27±0.01c |
|  |  | 20-40 | 0.35±0.05b | 0.37±0.09b | 0.36±0.08b | 0.29±0c |
|  |  | 40-60 | 1.24±0.56a | 1.09±0.54a | 0.45±0.09b | 0.39±0.01c |
|  | Pa-L | 0-20 | 1±0a | 1±0a | 1±0a | 1±0a |
|  |  | 20-40 | 1±0a | 1±0a | 1±0a | 1±0a |
|  |  | 40-60 | 1±0a | 1±0a | 1±0a | 1±0a |
|  | Pa-M | 0-20 | 0.08±0b | 0.07±0b | 0.07±0.01b | 0.15±0.01b |
|  |  | 20-40 | 0.17±0.01b | 0.2±0.03b | 0.22±0.03b | 0.24±0.01b |
|  |  | 40-60 | 0.41±0.07b | 0.37±0.05b | 0.39±0.08b | 0.44±0.01b |
|  | Pa-N | 0-20 | 0.04±0.01c | 0.04±0.01c | 0.04±0c | 0.05±0c |
|  |  | 20-40 | 0.11±0.02c | 0.12±0.02c | 0.11±0.01c | 0.11±0c |
|  |  | 40-60 | 0.36±0.05b | 0.37±0.05b | 0.35±0.05b | 0.29±0.01c |
|  | Pt-L | 0-20 | 1±0a | 1±0a | 1±0a | 1±0a |
|  |  | 20-40 | 1±0a | 1±0a | 1±0a | 1±0a |
|  |  | 40-60 | 1±0a | 1±0a | 1±0a | 1±0a |
|  | Pt-M | 0-20 | 0.25±0.05b | 0.24±0.05b | 0.29±0.07b | 0.53±0.06b |
|  |  | 20-40 | 0.22±0.04b | 0.24±0.02c | 0.28±0.04c | 0.45±0.02b |
|  |  | 40-60 | 0.42±0.04b | 0.46±0.05b | 0.45±0.11b | 0.7±0.06b |
|  | Pt-N | 0-20 | 0.05±0c | 0.06±0c | 0.11±0.01c | 0.12±0c |
|  |  | 20-40 | 0.4±0.11b | 0.5±0.11b | 0.5±0.09b | 0.41±0.01b |
|  |  | 40-60 | 0.36±0.08b | 0.41±0.06b | 0.47±0.08b | 0.34±0.02c |

**Figure S8A-8C.** Vertical distribution ratio of graded root surface area of three conifers(data in Figure 8A-8C)

| Indicator | Serial number | Soil depth (cm) | The ratio of the transport root(＞6mm)(%) | The ratio of the absorption root(≤6mm)(%) |
| --- | --- | --- | --- | --- |
| The ratio of the surface area of the root(%) | Pa-L | 0-20 | 53.61±1.04a | 4.35±0.2a |
|  |  | 20-40 | 25.84±0.89b | 1.48±0.07a |
|  |  | 40-60 | 14±0.52c | 0.73±0.08b |
|  | Pa-M | 0-20 | 37.09±0.28b | 1.41±0.11b |
|  |  | 20-40 | 29.24±0.16b | 1.47±0.15a |
|  |  | 40-60 | 29.44±0.3b | 1.35±0.29b |
|  | Pa-N | 0-20 | 26.56±0.41c | 1.62±0.19b |
|  |  | 20-40 | 27.26±0.26a | 1.68±0.11a |
|  |  | 40-60 | 40.35±0.12a | 2.53±0.18a |
|  | Pt-L | 0-20 | 38.97±0.31a | 3.46±0.14a |
|  |  | 20-40 | 30.95±0.76b | 2.62±0.18b |
|  |  | 40-60 | 22.46±0.75b | 1.54±0.15b |
|  | Pt-M | 0-20 | 38.84±1.6a | 1.78±0.33b |
|  |  | 20-40 | 26.85±2.23b | 1.36±0.24b |
|  |  | 40-60 | 29.85±0.57a | 1.33±0.29b |
|  | Pt-N | 0-20 | 17.51±0.4b | 1.12±0.13b |
|  |  | 20-40 | 46±0.76a | 4.68±0.68a |
|  |  | 40-60 | 28.24±0.23a | 2.45±0.19a |
|  | Pb-L | 0-20 | 43.33±1.11a | 3.26±0.9a |
|  |  | 20-40 | 27.21±0.87b | 1.56±0.15a |
|  |  | 40-60 | 23.55±0.96c | 1.08±0.1a |
|  | Pb-M | 0-20 | 37.26±0.46b | 2.67±0.49a |
|  |  | 20-40 | 29.84±0.65a | 1.81±0.52a |
|  |  | 40-60 | 27.2±0.31b | 1.22±0.36a |
|  | Pb-N | 0-20 | 38.38±0.56b | 2.52±0.41a |
|  |  | 20-40 | 25.39±0.38b | 1.82±0.42a |
|  |  | 40-60 | 29.87±0.48a | 2±0.6a |

**Figure S8D-8F.** The percentage of root surface area in the vertical direction of soil for three conifers(data in Figure 8D-8F)

| Indicator | Serial number | Soil depth(cm) | | |
| --- | --- | --- | --- | --- |
|  |  | 0-20 | 20-40 | 40-60 |
| The ratio of the surface area of the root(%) | Pa-L | 57.95±0.84a | 27.31±0.9b | 14.73±0.48c |
|  | Pa-M | 38.5±0.24b | 30.71±0.14a | 30.8±0.16b |
|  | Pa-N | 28.18±0.24c | 28.94±0.34ab | 42.88±0.14a |
|  | Pt-L | 42.44±0.22a | 33.57±0.67b | 23.99±0.84b |
|  | Pt-M | 40.61±1.91a | 28.21±2.45c | 31.18±0.54a |
|  | Pt-N | 18.64±0.3b | 50.68±0.1a | 30.68±0.31a |
|  | Pb-L | 46.59±1.97a | 28.78±1.02a | 24.63±0.96c |
|  | Pb-M | 39.93±0.16b | 31.65±0.14b | 28.42±0.13b |
|  | Pb-N | 40.91±0.19b | 27.22±0.07b | 31.88±0.2c |

**Figure S9A-11C.** Distribution of root volume in the vertical and horizontal directions of three coniferous species(data in Figure 9A-11C)

| Indicator | Serial number | Soil depth(cm) | The horizontal distance of the soil (cm） | | | | | | | | |
| --- | --- | --- | --- | --- | --- | --- | --- | --- | --- | --- | --- |
|  |  |  | A(20-40) | B(40-60) | C(60-80) | D(80-100) | E(100-120) | F(120-140) | G(140-160) | H(160-180) | I(180-200) |
| The volume of the root(cm^3^) | Pt-L | 0-20 | 31.55±0.75a | 36.57±0.56a | 26.68±2.02a | 14.48±0.1 | 4.72±0.86 |  |  |  |  |
|  |  | 20-40 | 22.33±1.57a | 22.9±1.96a | 18.5±0.1a | 10.5±0.69 | 2.84±0.48 |  |  |  |  |
|  |  | 40-60 | 18.45±0.24a | 19.38±0.44b | 12.04±0.21a | 6.91±1.42 |  |  |  |  |  |
|  | Pt-M | 0-20 | 16.32±0.64b | 26.4±1.79b | 17.19±0.87b |  |  |  |  |  |  |
|  |  | 20-40 | 11.8±0.28b | 22.3±0.92a | 9.71±1.57b |  |  |  |  |  |  |
|  |  | 40-60 | 12.63±2.82b | 23.04±0.88a | 12.17±0.09a |  |  |  |  |  |  |
|  | Pt-N | 0-20 | 4.46±0.52c | 4.92±0.79c | 1.91±1.06c |  |  |  |  |  |  |
|  |  | 20-40 | 9.12±0.29b | 10.52±0.4b | 7.17±0.06b |  |  |  |  |  |  |
|  |  | 40-60 | 7.89±0.26b | 12.64±0.92c | 5.93±0.48b |  |  |  |  |  |  |
|  | Pa-L | 0-20 | 38.84±0.02a | 65.98±7.34a | 69.74±4.21a | 71.99±0.68a | 54.84±1.08 | 22.39±0.83 | 11.2±1.19 | 7.3±0.64 | 4.53±1.13 |
|  |  | 20-40 | 30.82±3.37a | 41.4±6.04a | 55.28±4.61a | 56.9±0.63 | 27.89±1.13 | 6.22±0.61 | 5.01±0.63 | 3.59±0.29 |  |
|  |  | 40-60 | 22±1.15a | 22.65±1.63a | 21.71±0.85a |  |  |  |  |  |  |
|  | Pa-M | 0-20 | 18.69±0.3b | 17.22±0.91b | 12.37±0.46b | 6.91±1.75b |  |  |  |  |  |
|  |  | 20-40 | 13.55±0.32b | 10.83±0.23b | 9.64±0.45b | 5.12±0.35 |  |  |  |  |  |
|  |  | 40-60 | 14.41±0.22b | 13.42±0.55b | 10.86±0.23b |  |  |  |  |  |  |
|  | Pa-N | 0-20 | 6.35±0.02c | 4.63±0.13b | 3.79±0.25c | 3.77±0.4c |  |  |  |  |  |
|  |  | 20-40 | 7.22±0.53b | 5.73±0.08b | 4.46±0.15b |  |  |  |  |  |  |
|  |  | 40-60 | 13.82±0.12b | 7.03±0.27c | 5.59±0.21c |  |  |  |  |  |  |
|  | Pb-L | 0-20 | 55.19±2.4a | 59.92±6.01a | 37.17±0.23a |  |  |  |  |  |  |
|  |  | 20-40 | 35.57±0.83a | 44.36±2.99a | 28.88±0.88a |  |  |  |  |  |  |
|  |  | 40-60 | 27.34±0.9a | 22.61±0.03a | 15.73±0.2a |  |  |  |  |  |  |
|  | Pb-M | 0-20 | 18.17±0.51b | 23.96±0.5b | 17.02±0.63b |  |  |  |  |  |  |
|  |  | 20-40 | 15.2±0.58b | 20.47±0.33b | 11.18±0.45b |  |  |  |  |  |  |
|  |  | 40-60 | 14.54±1.11b | 18.84±0.72b | 10.95±0.29b |  |  |  |  |  |  |
|  | Pb-N | 0-20 | 10.99±0.42c | 11.56±0.77b | 7.92±0.31c |  |  |  |  |  |  |
|  |  | 20-40 | 12.09±1c | 13.29±0.36c | 8.86±0.54c |  |  |  |  |  |  |
|  |  | 40-60 | 12.55±0.69b | 12.29±0.23c | 8.07±0.02c |  |  |  |  |  |  |

**Figure S9D-9F.** Distribution of the volume of graded roots in the vertical direction in three coniferous species(data in Figure 9D-9F)

| Indicator | Serial number | Soil depth(cm) | Transport root (＞6mm) | Absorption root(≤6mm) |
| --- | --- | --- | --- | --- |
| The volume of the root(cm^3^) | Pa-L | 0-20 | 329.91±5.85a | 7.54±0.49a |
|  |  | 20-40 | 223.85±8.11a | 3.26±0.22a |
|  |  | 40-60 | 65.4±2.04a | 0.96±0.11a |
|  | Pa-M | 0-20 | 54.67±1.47b | 0.51±0.05b |
|  |  | 20-40 | 38.69±0.18b | 0.45±0.07b |
|  |  | 40-60 | 38.06±0.27b | 0.63±0.11a |
|  | Pa-N | 0-20 | 18.26±0.42c | 0.27±0.05b |
|  |  | 20-40 | 17.17±0.31c | 0.24±0.07b |
|  |  | 40-60 | 25.74±0.32c | 0.69±0.26a |
|  | Pt-L | 0-20 | 111.27±1.42a | 2.72±0.14a |
|  |  | 20-40 | 75.25±0.98a | 1.84±0.19a |
|  |  | 40-60 | 55.71±1.82a | 1.07±0.1a |
|  | Pt-M | 0-20 | 58.88±2.77b | 1.03±0.22b |
|  |  | 20-40 | 43.37±2.06b | 0.45±0.13b |
|  |  | 40-60 | 47.69±2.93b | 0.54±0.12b |
|  | Pt-N | 0-20 | 11.03±1.23c | 0.26±0.02c |
|  |  | 20-40 | 26.04±0.69c | 0.77±0.04b |
|  |  | 40-60 | 25.86±0.97c | 0.6±0.06c |
|  | Pb-L | 0-20 | 149.65±8.67a | 2.63±0.28a |
|  |  | 20-40 | 107.09±3.53a | 1.73±0.24a |
|  |  | 40-60 | 64.67±1.01a | 1.01±0.01a |
|  | Pb-M | 0-20 | 57.76±1.66b | 1.39±0.29b |
|  |  | 20-40 | 45.91±0.17b | 0.93±0.35a |
|  |  | 40-60 | 43.11±1.78b | 0.59±0.23a |
|  | Pb-N | 0-20 | 32.26±2.2c | 0.67±0.1b |
|  |  | 20-40 | 30.65±1.38c | 0.82±0.36a |
|  |  | 40-60 | 31.79±0.66c | 0.67±0.11a |

**Figure S9G-9I.** The ratio of graded root volume to grade 1 in the vertical direction in three coniferous species(data in Figure 9G-9I)

| Indicator | Serial number | Soil depth(cm) | Grading of root system(mm) | | | |
| --- | --- | --- | --- | --- | --- | --- |
|  |  |  | I(＜2) | Ⅱ(2-4) | Ⅲ(4-6) | Ⅳ(＞6) |
| The ratio of graded root volume to grade 1 | Pb-L | 0-20 | 1.00±0.00a | 1.00±0.00a | 1.00±0.00a | 1.00±0.00a |
|  |  | 20-40 | 1.00±0.00a | 1.00±0.00a | 1.00±0.00a | 1.00±0.00a |
|  |  | 40-60 | 1.00±0.00a | 1.00±0.00a | 1.00±0.00a | 1.00±0.00a |
|  | Pb-M | 0-20 | 0.46±0.07b | 0.63±0.16b | 0.55±0.17b | 0.39±0.03b |
|  |  | 20-40 | 0.36±0.10a | 0.51±0.19a | 0.56±0.21a | 0.43±0.01b |
|  |  | 40-60 | 0.45±0.09a | 0.84±0.23a | 0.55±0.23a | 0.67±0.04b |
|  | Pb-N | 0-20 | 0.31±0.05b | 0.29±0.03b | 0.25±0.01b | 0.22±0.01c |
|  |  | 20-40 | 0.74±0.32a | 0.79±0.50a | 0.5±0.27a | 0.29±0.01c |
|  |  | 40-60 | 0.94±0.39a | 1.00±0.36a | 0.62±0.10a | 0.49±0.02c |
|  | Pa-L | 0-20 | 1.00±0.00a | 1.00±0.00a | 1.00±0.00a | 1.00±0.00a |
|  |  | 20-40 | 1.00±0.00a | 1.00±0.00a | 1.00±0.00a | 1.00±0.00a |
|  |  | 40-60 | 1.00±0.00a | 1.00±0.00a | 1.00±0.00a | 1.00±0.00a |
|  | Pa-M | 0-20 | 0.09±0.01b | 0.07±0.00b | 0.07±0.00b | 0.17±0.00b |
|  |  | 20-40 | 0.15±0.01b | 0.15±0.01b | 0.14±0.02b | 0.17±0.01b |
|  |  | 40-60 | 0.55±0.03a | 0.58±0.11a | 0.71±0.16a | 0.58±0.01b |
|  | Pa-N | 0-20 | 0.04±0.01c | 0.04±0.01b | 0.04±0.01c | 0.06±0.00c |
|  |  | 20-40 | 0.06±0.01c | 0.07±0.01c | 0.07±0.03b | 0.08±0.00c |
|  |  | 40-60 | 0.99±0.51a | 0.96±0.51a | 0.73±0.30a | 0.39±0.02c |
|  | Pt-L | 0-20 | 1.00±0.00a | 1.00±0.00a | 1.00±0.00a | 1.00±0.00a |
|  |  | 20-40 | 1.00±0.00a | 1.00±0.00a | 1.00±0.00a | 1.00±0.00a |
|  |  | 40-60 | 1.00±0.00a | 1.00±0.00a | 1.00±0.00a | 1.00±0.00a |
|  | Pt-M | 0-20 | 0.39±0.16b | 0.35±0.09b | 0.4±0.11b | 0.53±0.02b |
|  |  | 20-40 | 0.31±0.14b | 0.26±0.09b | 0.26±0.09b | 0.58±0.03b |
|  |  | 40-60 | 0.6±0.16ab | 0.61±0.14b | 0.5±0.15ab | 0.86±0.04b |
|  | Pt-N | 0-20 | 0.05±0.01b | 0.07±0.00c | 0.11±0.00c | 0.1±0.01c |
|  |  | 20-40 | 0.40±0.13b | 0.46±0.12b | 0.42±0.02b | 0.35±0.01b |
|  |  | 40-60 | 0.40±0.10b | 0.48±0.1b | 0.62±0.12b | 0.47±0.03c |

**Figure S10A-10C.** Vertical distribution ratio of graded root volume of three conifers(data in Figure 10A-10C)

| Indicator | Serial number | Soil depth(cm) | Transport root (＞6mm) | Absorption root(≤6mm) |
| --- | --- | --- | --- | --- |
| The ratio of the volume of the root(%) | Pa-L | 0-20 | 52.3±0.91a | 1.20±0.09a |
|  |  | 20-40 | 35.47±1.02a | 0.52±0.04a |
|  |  | 40-60 | 10.36±0.2c | 0.14±0.01b |
|  | Pa-M | 0-20 | 41.09±0.59b | 0.38±0.04b |
|  |  | 20-40 | 29.09±0.22b | 0.34±0.05a |
|  |  | 40-60 | 28.62±0.28b | 0.48±0.09ab |
|  | Pa-N | 0-20 | 29.27±0.58c | 0.44±0.08b |
|  |  | 20-40 | 27.52±0.41b | 0.38±0.11a |
|  |  | 40-60 | 41.28±0.71a | 1.11±0.40a |
|  | Pt-L | 0-20 | 44.89±0.45a | 1.1±0.05a |
|  |  | 20-40 | 30.36±0.45b | 0.74±0.08b |
|  |  | 40-60 | 22.48±0.70c | 0.43±0.04b |
|  | Pt-M | 0-20 | 38.73±1.17b | 0.69±0.17b |
|  |  | 20-40 | 28.52±0.70b | 0.30±0.08c |
|  |  | 40-60 | 31.42±1.88b | 0.35±0.07b |
|  | Pt-N | 0-20 | 17.00±1.28c | 0.41±0.04b |
|  |  | 20-40 | 40.4±1.22a | 1.21±0.1a |
|  |  | 40-60 | 40.06±0.22a | 0.93±0.09a |
|  | Pb-L | 0-20 | 45.75±1.9a | 0.81±0.1a |
|  |  | 20-40 | 32.8±1.36a | 0.53±0.07a |
|  |  | 40-60 | 19.8±0.44c | 0.31±0a |
|  | Pb-M | 0-20 | 38.44±1.27b | 0.92±0.2a |
|  |  | 20-40 | 30.54±0.19a | 0.62±0.23a |
|  |  | 40-60 | 28.67±0.98b | 0.40±0.15a |
|  | Pb-N | 0-20 | 32.82±1.87b | 0.69±0.11a |
|  |  | 20-40 | 31.20±1.06a | 0.85±0.38a |
|  |  | 40-60 | 32.41±1.05a | 0.68±0.12a |

**Figure S10D-10F.** The percentage of root volume in the vertical direction of soil for three conifers(data in Figure 10D-10F)

| Indicator | Serial number | Soil depth(cm) | | |
| --- | --- | --- | --- | --- |
|  |  | 0-20 | 20-40 | 40-60 |
| The ratio of the volume of the root(%) | Pa-L | 53.5±0.96a | 35.99±1.01a | 10.51±0.20c |
|  | Pa-M | 41.47±0.57b | 29.43±0.28b | 29.1±0.31b |
|  | Pa-N | 29.71±0.51c | 27.91±0.36b | 42.39±0.41a |
|  | Pt-L | 45.99±0.42a | 31.1±0.41b | 22.91±0.73c |
|  | Pt-M | 39.42±1.11b | 28.81±0.75b | 31.77±1.83b |
|  | Pt-N | 17.4±1.26c | 41.61±1.29a | 40.99±0.14a |
|  | Pb-L | 46.56±1.84a | 33.33±1.40a | 20.11±0.44c |
|  | Pb-M | 39.36±1.25b | 31.16±0.35a | 29.48±1.19b |
|  | Pb-N | 34.86±0.53b | 32.05±0.69a | 33.1±1.17a |

**Figure S11A-11C.** Effects of high soil water content on root activity of three coniferous species with graded roots (data in Figure S11A-11C)

| Indicator | Soil depth(cm) | Serial number | Grading of root system(mm) | | | |
| --- | --- | --- | --- | --- | --- | --- |
|  |  |  | I(＜2) | Ⅱ(2-4) | Ⅲ(4-6) | Ⅳ(＞6) |
| The root activity(ug/(kg·h)) | 0-20 | Pb-L | 35.49±0.39a | 24.53±0.27a | 20.31±0.18a | 18.15±0.37a |
|  |  | Pb-M | 22.19±0.34b | 20.3±0.12b | 18.22±0.39b | 16.13±0.37b |
|  |  | Pb-N | 16.46±3.17c | 13.34±0.25c | 11.21±0.21c | 10.03±0.3c |
|  | 20-40 | Pb-L | 37.34±0.15a | 29.06±0.13a | 22.25±0.24a | 20.17±0.43a |
|  |  | Pb-M | 23.77±0.21b | 21.1±0.24b | 19.4±0.18b | 15.31±0.18b |
|  |  | Pb-N | 17.35±0.28c | 15.97±0.23c | 13.64±0.25c | 11.69±0.25c |
|  | 40-60 | Pb-L | 21.04±0.21a | 19.75±0.19a | 16.14±0.42a | 11.83±0.32a |
|  |  | Pb-M | 21.97±0.57b | 17.33±0.22b | 13.78±0.18b | 10.17±0.32b |
|  |  | Pb-N | 13.43±0.96c | 5.58±0.24c | 4.26±0.5c | 2.53±0.18c |
|  | 0-20 | Pa-L | 36.96±0.14a | 29.49±0.3a | 33.37±0.14a | 28.29±0.25a |
|  |  | Pa-M | 32.54±0.24b | 24.8±0.42b | 22.89±0.47b | 16.7±0.28b |
|  |  | Pa-N | 26.28±0.42c | 20.79±0.27c | 18.94±0.29c | 13.57±0.42c |
|  | 20-40 | Pa-L | 38.57±0.21a | 34.67±0.24a | 28.84±0.17a | 26.86±0.23a |
|  |  | Pa-M | 31.00±0.53b | 26±0.12b | 24.47±0.5b | 21.9±0.18b |
|  |  | Pa-N | 30.78±0.55c | 23.17±0.84c | 19.56±0.23c | 15.35±1.12c |
|  | 40-60 | Pa-L | 28.48±0.36a | 25.65±0.25a | 24.98±0.13a | 19.7±0.51a |
|  |  | Pa-M | 18.78±0.32b | 14.68±0.19b | 13.58±0.31b | 13.31±0.25b |
|  |  | Pa-N | 20.57±0.15c | 13.57±0.24c | 9.33±0.12c | 6.14±0.25c |
|  | 0-20 | Pt-L | 37.46±0.32a | 35.73±0.42a | 34.92±0.5a | 32.81±1.35a |
|  |  | Pt-M | 30.68±0.27b | 28.39±0.76b | 27.62±0.31b | 25.54±0.23b |
|  |  | Pt-N | 19.75±0.35c | 18.05±0.53c | 17.44±0.9c | 15.05±0.34c |
|  | 20-40 | Pt-L | 39.06±0.30a | 36.70±0.44a | 33.62±0.39a | 32.71±0.15a |
|  |  | Pt-M | 31.89±0.35b | 29.40±0.32b | 28.27±0.2b | 27.37±0.33b |
|  |  | Pt-N | 21.02±0.23c | 19.84±0.32c | 18.38±0.2c | 13.43±0.27c |
|  | 40-60 | Pt-L | 27.27±1.92a | 23.41±2.39a | 18.90±0.34a | 15.44±0.43a |
|  |  | Pt-M | 23.38±0.14b | 17.20±0.42b | 15.21±0.17b | 11.69±0.40b |
|  |  | Pt-N | 14.57±0.72c | 12.11±1.42b | 9.40±0.33c | 8.13±0.22c |

**Figure S11D-11F.** Root activity of the three conifer root classes varied in the vertical direction of soil(data in Figure 11D-11F)

| Indicator | Serial number | Soil depth(cm) | | |
| --- | --- | --- | --- | --- |
|  |  | 0-20 | 20-40 | 40-60 |
| The root activity(ug/(kg·h)) | Pa-L | 32.35±0.41a | 33.51±0.52a | 25.91±0.39a |
|  | Pa-M | 27.66±0.77b | 29.30±1.05b | 16.40±0.6b |
|  | Pa-N | 21.11±0.63c | 22.89±0.91c | 15.20±0.72b |
|  | Pt-L | 35.36±0.38a | 36.70±0.35a | 21.71±1.10a |
|  | Pt-M | 28.76±0.35b | 29.46±0.38b | 16.87±0.73b |
|  | Pt-N | 18.05±0.31c | 19.19±0.34c | 11.47±0.52c |
|  | Pb-L | 28.66±0.94a | 29.62±1.02a | 19.38±0.56a |
|  | Pb-M | 20.74±0.29b | 21.96±0.42b | 17.48±0.59a |
|  | Pb-N | 13.78±1.11c | 16.14±0.41c | 8.32±1.02b |

**Figure S12A-12C.** Effects of high soil water content on root respiration of three coniferous species with graded roots(data in Figure 12A-12C)

| Indicator | Soil depth(cm) | Serial number | Grading of root system(mm) | | | |
| --- | --- | --- | --- | --- | --- | --- |
|  |  |  | I(＜2) | Ⅱ(2-4) | Ⅲ(4-6) | Ⅳ(＞6) |
| The root respiration(mg/kg·h) | 0-20 | Pa-L | 2102.22±12.93a | 1598.67±29.09a | 1566.07±30.41a | 1223.62±29.13a |
|  |  | Pa-M | 1354.22±46.64b | 1315.11±40.02b | 1266.83±9.97b | 1065.78±21.86b |
|  |  | Pa-N | 1212.44±138.8c | 1144.±30.53c | 1111.41±13.99c | 898.86±27.29c |
|  | 20-40 | Pa-L | 2366.22±25.87a | 2278.22±17.63a | 1749.00±19.35a | 1408.00±10.71a |
|  |  | Pa-M | 1719.67±34.98b | 1683.00±16.80b | 1549.78±39.66b | 1300.44±36.06b |
|  |  | Pa-N | 1280.89±40.02c | 1075.56±29.74c | 968.00±37.39c | 909.33±54.88c |
|  | 40-60 | Pa-L | 2107.13±48.15a | 1984.891±9.56a | 1334.67±81.88a | 1148.89±27.74a |
|  |  | Pa-M | 1300.4±56.38b | 1207.56±24.44b | 1102.1±21.09b | 987.56±36.85b |
|  |  | Pa-N | 1114.67±25.40c | 1036.45±43.45c | 914.22±29.5c | 815.83±50.19c |
|  | 0-20 | Pb-L | 1584.00±55.53a | 1544.89±25.87a | 1410.1±31.10a | 1259.5±13.70a |
|  |  | Pb-M | 1403.11±39.11b | 1378.67±38.8b | 1301.67±27.12b | 1095.11±14.98b |
|  |  | Pb-N | 1160.76±27.48c | 1068.57±29.03 | 987.56±27.17c | 834.37±24.50c |
|  | 20-40 | Pb-L | 1852.89±19.56a | 1760.00±33.87a | 1499.67±33.35a | 1349.33±31.36a |
|  |  | Pb-M | 1628.00±50.81b | 1427.56±46.64b | 1287.733±32.93b | 1214.4±33.25b |
|  |  | Pb-N | 1339.56±12.93c | 1256.44±42.62c | 1188.00±13.61c | 1047.85±17.30 |
|  | 40-60 | Pb-L | 1324.89±21.31a | 1236.89±29.74a | 1126.4±38.92a | 953.33±33.26a |
|  |  | Pb-M | 1227.11±12.93b | 1114.67±36.91b | 947.14±40.58 | 809.11±43.92b |
|  |  | Pb-N | 1056.00±22.4c | 928.89±29.73c | 829.48±26.49c | 697.48±132.63c |
|  | 0-20 | Pt-L | 2112.00±25.12a | 1826.00±12.7a | 1659.42±31.27a | 1478.07±37.53a |
|  |  | Pt-M | 1760.00±55.53b | 1628.00±8.47b | 1225.48±12.73b | 989.190±19.26b |
|  |  | Pt-N | 1188.00±33.87c | 1056.00±22.4c | 917.48±16.60c | 749.63±105.27c |
|  | 20-40 | Pt-L | 2361.33±8.47a | 2244.00±8.47a | 2009.3±18.93a | 1811.33±26.87 |
|  |  | Pt-M | 1823.56±40.02b | 1745.33±59.27b | 1549.78±29.74b | 1063.3±25.61b |
|  |  | Pt-N | 1549.78±25.87c | 1412.89±25.87c | 1357.71±20.71c | 875.11±80.74c |
|  | 40-60 | Pt-L | 1271.11±21.31a | 1153.78±51.04a | 1009.56±34.17a | 862.89±35a |
|  |  | Pt-M | 1065.78±29.74b | 928.89±43.45b | 865.33±52.88b | 718.67±46.69b |
|  |  | Pt-N | 938.67±25.40c | 738.22±48.15c | 689.3±49.74c | 575.26±27.74c |

**Figure S12D-12.** Root respiration of the three conifer root classes varied in the vertical direction of soil (data in Figure 12D-12F)

| Indicator |  | Soil depth(cm) | | |
| --- | --- | --- | --- | --- |
|  | Serial number | 0-20 | 20-40 | 40-60 |
| The root respiration(mg/kg·h) | Pa-L | 1542.35±54.84a | 1878.61±79.18a | 1484.83±88.83a |
|  | Pa-M | 1205.86±27.07b | 1517.19±43.87b | 1126.67±27.39b |
|  | Pa-N | 1062.00±27.49c | 1028.5±36.49c | 923.27±33.39c |
|  | Pt-L | 1726.72±50.86a | 2019.11±54.06a | 1028.3±39.63a |
|  | Pt-M | 1254.00±58.41b | 1465.85±74.74b | 859.47±40.53b |
|  | Pt-N | 905.67±49.54c | 1194.00±67.77c | 682.73±33.74c |
|  | Pb-L | 1396.83±30.73a | 1528.67±43.31a | 1102.32±37.61a |
|  | Pb-M | 1266.47±30.18b | 1354.83±42.48b | 971.63±32.84b |
|  | Pb-N | 961.89±27.29c | 1162.94±22.98c | 820.72±30.55c |
