# Supplementary figures and images for: Study on the causes of growth differences in three conifers after the rainy season in the Xiong’an New Area
Source: Front Plant Sci. 2023 Jul 4;14:1176142. doi: 10.3389/fpls.2023.1176142 (PMC10352786; doi:10.3389/fpls.2023.1176142)

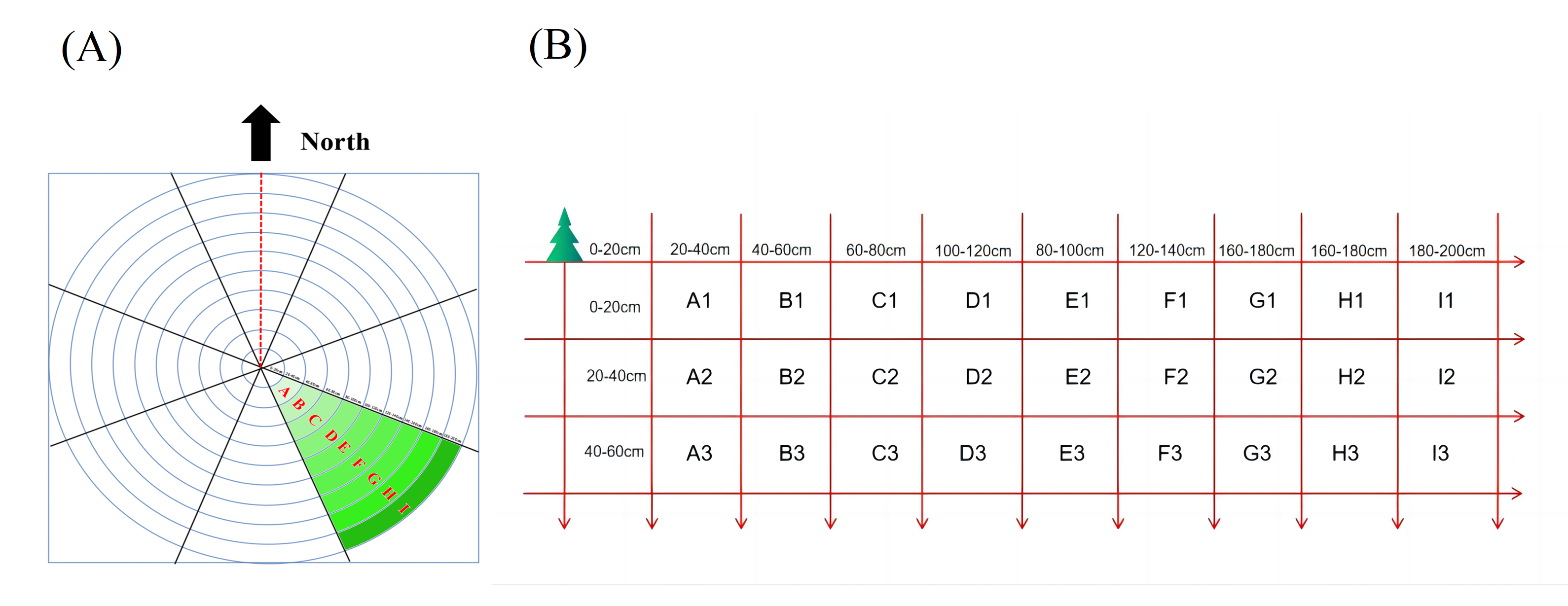

Supplement: Supplementary file 2 [file Image_1.tif]

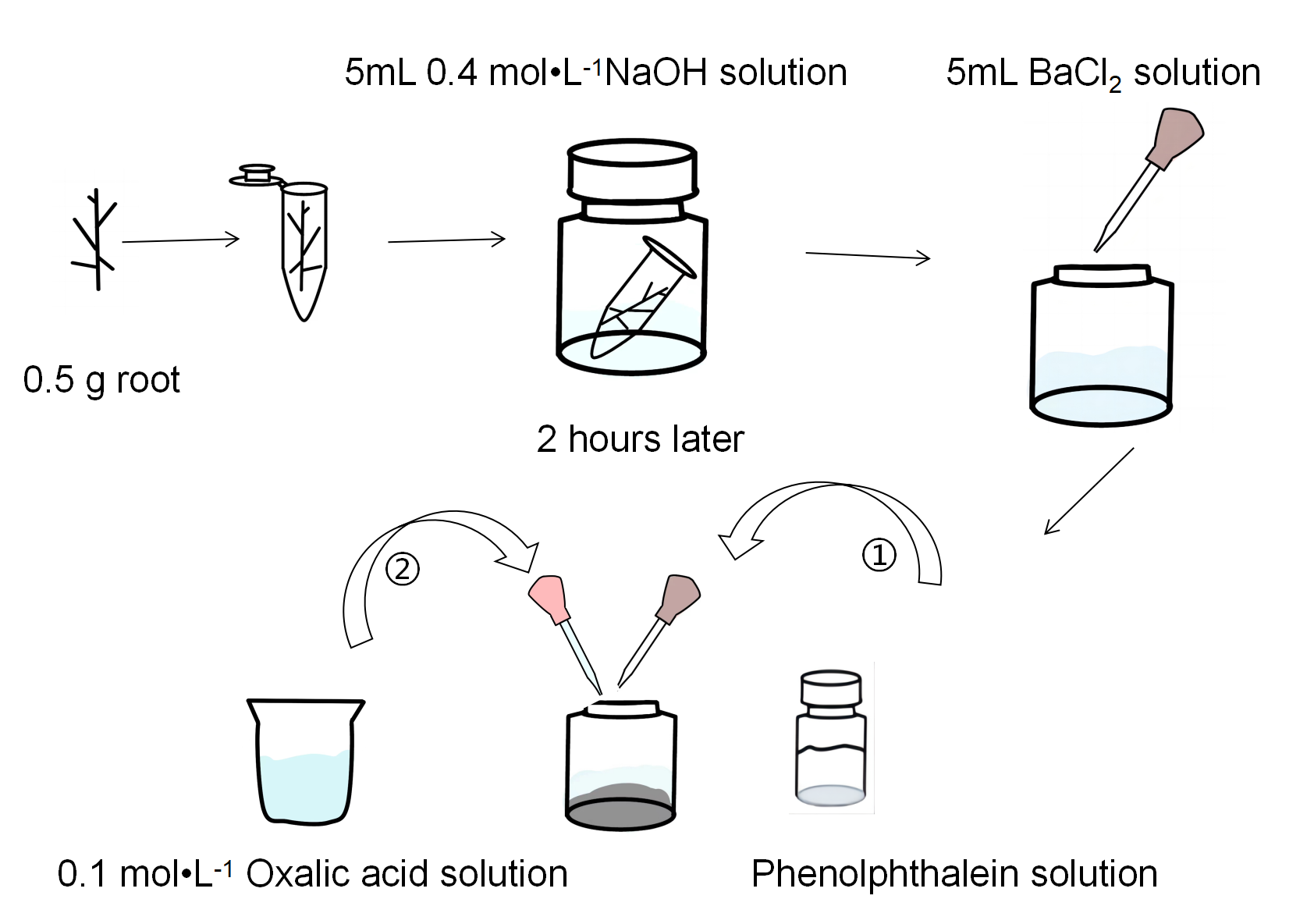

Supplement: Supplementary file 3 [file Image_2.tif]

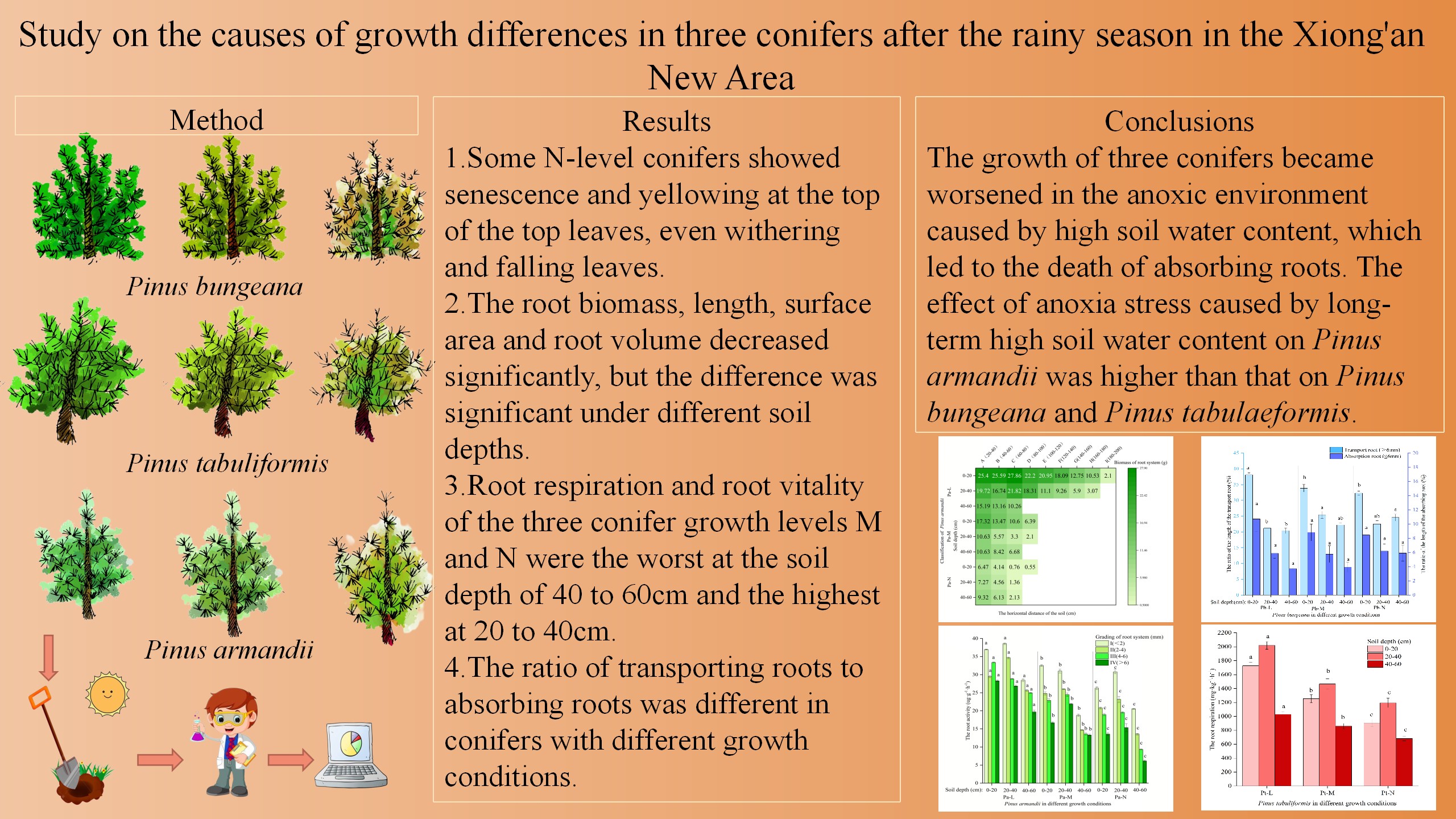

Supplement: Supplementary file 4 [file Image_3.jpg]
